# Silibinin inhibits PM2.5-induced liver triglyceride accumulation through enhancing the function of mitochondrial Complexes I and II

**DOI:** 10.3389/fphar.2024.1435230

**Published:** 2024-09-16

**Authors:** Dexin Li, Jingxin Zhang, Yuxin Jin, Yaoxuan Zhu, Xiaoqing Lu, Xinmei Huo, Chunshui Pan, Lijun Zhong, Kai Sun, Li Yan, Lulu Yan, Ping Huang, Quan Li, Jing-Yan Han, Yin Li

**Affiliations:** ^1^ Department of Integration of Chinese and Western Medicine, School of Basic Medical Sciences, Peking University, Beijing, China; ^2^ Tasly Microcirculation Research Center, Peking University Health Science Center, Beijing, China; ^3^ The Key Discipline for Integration of Chinese and Western Basic Medicine (Microcirculation) of the National Administration of Traditional Chinese Medicine, Beijing, China; ^4^ Key Laboratory of Stasis and Phlegm, State Administration of Traditional Chinese Medicine of the People’s Republic of China, Beijing, China; ^5^ Beijing Microvascular Institute of Integration of Chinese and Western Medicine, Beijing, China; ^6^ Peking University Medical and Health Analysis Center, Peking University, Beijing, China

**Keywords:** silibinin, lipid accumulation, PM2.5, mitochondrial dysfunction, oxidative stress

## Abstract

**Background:**

The standardized extract of milk thistle seeds, known as silibinin, has been utilized in herbal medicine for over two centuries, with the aim of safeguarding the liver against the deleterious effects of various toxic substances. However, the role of silibinin in Particulate Matter (PM2.5)-induced intrahepatic triglyceride accumulation remains unclear. This study seeks to investigate the impact of silibinin on PM2.5-induced intrahepatic triglyceride accumulation and elucidate potential underlying mechanisms.

**Methods:**

A model of intrahepatic triglyceride accumulation was established in male C57BL/6J mice through intratracheal instillation of PM2.5, followed by assessment of liver weight, body weight, liver index, and measurements of intrahepatic triglycerides and cholesterol after treatment with silibinin capsules. Hep G2 cells were exposed to PM2.5 suspension to create an intracellular triglyceride accumulation model, and after treatment with silibinin, cell viability, intracellular triglycerides and cholesterol, fluorescence staining for Nile Red (lipid droplets), and DCFH-DA (Reactive Oxygen Species, ROS), as well as proteomics, real-time PCR, and mitochondrial function assays, were performed to investigate the mechanisms involved in reducing triglycerides.

**Results:**

PM2.5 exposure leads to triglyceride accumulation, increased ROS production, elevated expression of inflammatory factors, decreased expression of antioxidant factors, and increased expression of downstream genes of aryl hydrocarbon receptor. Silibinin can partially or fully reverse these factors, thereby protecting cells and animal livers from PM2.5-induced damage. *In vitro* studies show that silibinin exerts its protective effects by preserving oxidative phosphorylation of mitochondrial complexes I and II, particularly significantly enhancing the function of mitochondrial complex II. Succinate dehydrogenase (mitochondrial complex II) is a direct target of silibinin, but silibinin A and B exhibit different affinities for different subunits of complex II.

**Conclusion:**

Silibinin improved the accumulation of intrahepatic triglycerides induced by PM2.5, and this was, at least in part, explained by an enhancement of oxidative phosphorylation in mitochondrial Complexes I and II.

## Graphical Abstract

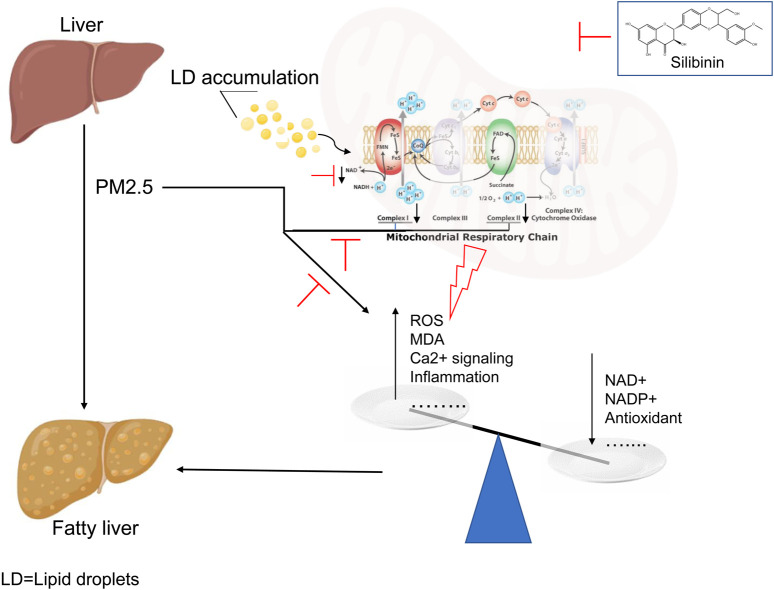

## Introduction

With the rapid development of the world economy, air pollution has become a significant threat to everyone’s health (Mohajeri et al., 2023). Among them, Particulate Matter (PM2.5) is the primary cause of air pollution and poses particularly severe hazards to the human body (Henning, 2024; Hou et al., 2023). Numerous studies both domestically and internationally have indicated that PM2.5 can penetrate deep into the lungs and enter the bloodstream, leading to cardiovascular and respiratory diseases (Fan et al., 2023; Sun et al., 2022). Among these, the liver, as the largest digestive gland and a central hub for metabolic activities in the human body, is significantly impacted. Exposure to a PM2.5 environment has been confirmed by numerous *in vivo* and *in vitro* experiments to induce the occurrence and development of metabolic-associated fatty liver disease (Ge et al., 2020; Xu et al., 2019).

Although extensive research has been conducted both domestically and internationally, yielding a series of advancements, the lack of effective drugs for treating metabolic-associated fatty liver disease persists (Mantovani et al., 2022). This is primarily due to potential differences in pathological mechanisms between the early and late stages of the disease, leading to the current absence of a definitive treatment (Chen et al., 2024; Parola and Pinzani, 2023). Recent studies have shown a close association between mitochondrial dysfunction and a spectrum of liver conditions, from simple steatosis to non-alcoholic steatohepatitis (Staňková et al., 2020; Guo et al., 2023). Mitochondria, essential organelles present in all nucleated cells, are known as the “powerhouses” of cells, responsible for energy production and regulating physiological and biochemical processes such as apoptosis, free radical generation, intracellular calcium homeostasis, and lipid metabolism. Dysfunction of mitochondria is associated with various diseases (Nguyen et al., 2023). Embedded on the inner membrane of mitochondria are four major respiratory chain complexes (also referred to as electron transport chain complexes), namely, Complex I, Complex II, Complex III, and Complex IV (Vercellino and Sazanov, 2022).

Silibinin, derived from the seeds of the annual or biennial thistle plant *Silybum marianum* (L.) Gaertn., has been successfully used as a natural herbal remedy for liver diseases for centuries (Yan et al., 2023; Takke and Shende, 2019). Functioning as a natural antioxidant, silibinin has been employed in the treatment of chronic liver diseases where the underlying pathogenic mechanisms are not clearly defined (Abenavoli et al., 2018). Notably, hepatoprotective characteristics are among the most common attributes associated with silibinin (Davis-Searles et al., 2005). Due to its cell-protective properties, silibinin has been used as an adjuvant in liver detoxification (Ma et al., 2024; Vecchione et al., 2016). However, whether silibinin exerts a beneficial effect on PM2.5-induced metabolism-related fatty liver disease and the associated mechanisms remain unclear. This study aims to investigate the role of silibinin in PM2.5-induced intrahepatic triglyceride accumulation and explore potential underlying mechanisms.

## Materials and methods

### Chemicals and reagents

PM2.5 particles (1650b) were procured from the National Institute of Standards and Technology (NIST) (MD, United States of America). Hep G2 cells were purchased from Pricella (Wuhan, China). The GPO Trinder enzymatic method was utilized for determining triglycerides (TG) and total cholesterol (TC) in cells and mouse liver, and the assay kits were obtained from Applygen (Beijing, China). Silibinin (S2357), used for cellular experiments, was acquired from Selleck (Shanghai, China). Silibinin Capsules (GuouaozhunziH20040299), used for animal experiments, were obtained from Tasly Pharmaceutical Co. Ltd. (Tianjin, China). Nile red and the Reactive Oxygen Species Assay Kit (DCFH-DA) were purchased from Applygen (Beijing, China). Tissue Superoxide Dismutase (SOD) Assay kit (WST-8 method), Tissue Total Glutathione Assay Kit, and Liquid Sample Malondialdehyde (MDA) Assay Kit (TBA method) were obtained from Applygen (Beijing, China). NAD/NADH Assay Kit and NADP/NADPH Assay Kit were purchased from Applygen (Beijing, China). Hieff^®^ qPCR SYBR Green Master Mix (No Rox) and Hifair^®^ Ⅲ first Strand cDNA Synthesis SuperMix for qPCR (gDNA digester plus) were procured from YEASEN (Shanghai, China). Silibinin A and Silibinin B were purchased from MCE (New Jersey, United States of America). The SDHA protein (CSB-YP020903HU) was obtained from CUSABIO Biotechnology Co., Ltd. (Wuhan, China). The SDHB protein (Ag29868) was sourced from Proteintech Biotechnology Co., Ltd. (Wuhan, China). Both SDHC (RPK213Mu01) and SDHD (RPK214Mu01) proteins were acquired from Cloud-Clone Corp (Wuhan, China).

### Preparation of PM2.5 suspension

Prior to usage, SRM 1650b (National Institute of Standards and Technology, NIST, MD, United States of America) was suspended in phosphate-buffered saline (PBS) at a concentration of 4 mg/mL. The suspension was vortexed, followed by 24 h of cumulative ultrasonication under ice bath conditions to disperse particles. The dispersed particles were then stored at 4°C in the dark for later use.

### Animal models

C57BL/6J mice, 8 weeks old, weighing 20–24 g, were purchased from Vital River Laboratory Animal Technology Co., Ltd. (Certificate No. SCXK (Jing) 2021–0075) and housed at the Experimental Animal Center of Peking University Health Science Center. Standard housing conditions were maintained (24°C ± 2°C, humidity 50% ± 5%, and a 12-h light-dark cycle). Animal experiments were conducted following the guidelines of the Experimental Animal Center and were approved by the Medicine Experimental Animal Research Ethics Committee of Peking University Health Science Center (Approval No. LA2018232). After acclimating for 1 week in standard conditions, all mice were randomly divided into four groups: (1) Negative control group (Control); (2) Drug group of silibinin Capsules (Silibinin); (3) PM2.5 exposure group (PM2.5); (4) Silibinin + PM2.5 group (Silibinin + PM2.5) (clinical equivalent dose 46.2 mg/kg). The drug dosage was determined based on pharmacological experimental methods (Nair et al., 2018), and further validated through experiments (Supplementary Figure S1A).

The negative control group (Control) and PM2.5 exposure group (PM2.5) were given the corresponding volume of saline by gavage. For mice exposed to PM2.5, the intratracheal dose was 12 mg/kgbw, administered once every 5 days for a total of 50 days. The negative control group (Control) and drug group of silibinin capsules (Silibinin) were given the corresponding volume of PBS by intratracheal administration. The method of PM2.5 intratracheal administration was consistent with the previously reported approach (Duan et al., 2022). In brief, after being anesthetized with 5% chloral hydrate, the mice were fixed. With the help of light, the pharyngeal region was located from the oral cavity of the mice. Then, the required volume of PM2.5 suspension was instilled into the trachea through a non-invasive method. After the mice regained consciousness, they were returned to their cages for rest.

### Cell culture and treatment

HepG2 cells were procured from Procell Life Science and Technology and cultured in DMEM medium (Invitrogen, NY, United States of America) supplemented with 10% fetal bovine serum (Invitrogen, NY, United States of America) and 100 U/mL penicillin-streptomycin at 37°C in a humidified atmosphere with 5% CO2. After reaching approximately 80% confluence, cells were subjected to specific treatments according to experimental requirements for a duration of 72 h.

### Cell viability assay

Assessment of cell viability was carried out using a Cell Counting Kit-8 (CCK-8), adhering to the manufacturer’s guidelines. In brief, 10 μL of CCK-8 solution (YEASEN, Shanghai, China) was introduced into each well, and the samples were incubated at 37°C for 2 h before measuring absorbance at a wavelength of 450 nm (Fu et al., 2024).

### Determination of TG and TC content in cells and liver tissues

The triglyceride and total cholesterol concentration was determined using the GPO-POD Triglyceride Assay Kit (Applygen, Beijing, China). In brief, Cells (1–2 × 10^6) were lysed and incubated with a working solution at 37°C for 10 min. Triglyceride concentration was measured at 550 nm using a microplate reader (Bio-Tek, Vermont, United States of America). The measurement of triglycerides and total cholesterol in mouse liver tissue is similar to the above steps (Luo et al., 2012).

### Nile red staining

The lipid content in HepG2 cells was determined using the Nile Red dye (Applygen, Beijing, China). In brief, cells were washed with serum-free DMEM, stained with Nile Red (1:500 dilution), and imaged using BioTek Cytation 7. Fluorescence values were recorded for analysis (Silva et al., 2023). In brief, we directly add the Nile Red fluorescent staining solution (500X) to the serum-free culture medium at a dilution ratio of 1:500, allow the cells to stain for 10 min, and then observe them under a microscope immediately, without the need to replace the medium or perform any washing steps.

### Intracellular ROS measurement

The intracellular generation of reactive oxygen species (ROS) was measured using the DCFH-DA probe method (Yang et al., 2024) (Applygen, Beijing, China). In brief, Hep G2 cells were incubated with a 5 μM DCFH-DA solution for 30 min. After washing and centrifugation, fluorescence was detected using BioTek Cytation 7.

### Determination of GSH, SOD and MDA levels

Glutathione (GSH), Superoxide dismutase (SOD), and Malondialdehyde (MDA) in cells was determined using the commercial assay kits (Beyotime, shanghai, China) following the manufacturer’s instructions (Wang and Zhang, 2024).

### Determination of NAD+/NADH and NADP+/NADPH levels in treated cells

Nicotinamide Adenine Dinucleotide, reduced form (NADH) and Nicotinamide Adenine Dinucleotide, (NAD+) levels in cells were measured using the NAD+/NADH assay kit with WST-8 (Applygen, Beijing, China). In brief, cell lysates were split into two fractions. One fraction was incubated to measure total NAD, while the other was heated to decompose NAD+. After adding detection reagent, absorbance at 450 nm was measured to determine NADH levels. NAD+/NADH ratio was calculated from total NAD and NADH levels. Additionally, measurements of NADP+/NADPH (Applygen, Beijing, China) were conducted following the manufacturer’s protocol, with steps similar to those used for measuring NAD+/NADH (Guo et al., 2024).

### Proteomics analysis

Tissue proteins extracted using lysis buffer were quantified and processed for mass spectrometry analysis. Trypsin digestion, data-dependent acquisition (DDA), and data-independent acquisition (DIA) were performed. PEAKS Online software analyzed acquired data. Functional and pathway analyses were conducted to understand biological events and identify key proteins (Cao et al., 2023).

### Real-time polymerase chain reaction

Total RNA was extracted from cells using Trizol reagent (Yeasen, Shanghai, China), and complementary cDNA was synthesized using the Hifair^®^ II First Strand cDNA Synthesis Kit (Yeasen, Shanghai, China). Real-time polymerase chain reaction analysis was performed on an Agilent AriaMx fluorescence quantitative PCR instrument (Agilent, Texas, United States of America) to measure the expression levels of inflammatory genes (Interleukin-1α (*IL-1*α), Interleukin-1β (*IL-1*β), NOD-Like Receptor Family, Pyrin Domain Containing 3 (*NLRP3*), Tumor Necrosis Factor-alpha (*TNF-*α), and Interleukin-8 (*IL-8*)), anti-inflammatory genes (Nuclear factor erythroid 2-related factor 2 (*Nrf2*), Heme oxygenase-1 (*HO-1*), NAD(P)H Quinone Oxidoreductase 1 (*NQO-1*), Glutamate-cysteine ligase catalytic subunit (*GCLC*) and Glutathione S-transferase (*GST*), and key genes in the oxidative phosphorylation pathway (Ubiquinol-Cytochrome c Reductase Binding Protein (*UQCRQ*), ATPase H + Transporting V0 Subunit C (*ATP6V0C*), Mitochondrial Complex I Subunit 8 (*NDUFA8*), ATPase H + Transporting V1 Subunit G2 (*ATP6V1G2*), Ubiquinol-Cytochrome c Reductase Core Protein 1 (*UQCRC1*), and Ubiquinol-Cytochrome c Reductase (*UQCRFS1*)). The primer sequences used in this study are listed in Supplementary Table S1.

### Mitochondrial respiration

Mitochondrial respiration was measured at 37°C using a high-resolution respirometry system (Jian et al., 2020) (Oxygraph-2k, Oroboros, Innsbruck, Austria). A total of 2 × 10^6 Hep G2 cells were suspended in 2 mL of MiR05 respiration buffer (20 mM taurine, 0.5 mM EGTA, 3 mM MgCl2·6H2O, 60 mM K-lactobionate, 10 mM KH2PO4, 20 mM HEPES, 110 mM D-sucrose, and 1 g/L BSA without fatty acids). In the protocol, digitonin (0.5 μg/mL) was added to permeabilize the cells. Sequential additions included substrates for complex I (pyruvate, malate, and glutamate - P + M + G at 5 mM, 2 mM, and 10 mM, respectively), ADP (2.5 mM), F1Fo ATP synthase inhibitor, oligomycin (2 μg/mL), mitochondrial uncoupler FCCP (0.5 μM/step until reaching maximum (OCR), and complex I inhibitor rotenone (0.5 μM) to assess complex I-related respiration. Further additions included rotenone (0.5 μM), complex II substrate succinate (5 mM), ADP (2.5 mM), oligomycin (2 μg/mL), FCCP (0.5 μM/step until maximum oxygen consumption rate (OCR), and complex III inhibitor antimycin A (2.5 μM) to evaluate complex II-related respiration. Lastly, the respiratory activity related to Complex IV was assessed by sequentially adding Complex III inhibitor antimycin A (2.5 μM), ascorbic acid and TMPD (As + Tm, at concentrations of 2 mM and 0.5 mM, respectively), ADP (2.5 mM), oligomycin (2 μg/mL), FCCP (0.5 μM/step until reaching maximum oxygen consumption rate (OCR). States of respiration were defined as follows: State one respiration: Oxygen consumption rate (OCR) at the start of the experiment without the addition of digitonin. State two respiration: oxygen consumption rate (OCR) upon addition of ADP before oligomycin, representing the maximum oxidative phosphorylation capacity (OXPHOS) for specific substrate combinations. State three respiration: oxygen consumption rate (OCR) after addition of oligomycin, representing LEAK respiration. State four respiration: Maximum oxygen consumption rate (OCR) under titration of FCCP, representing the maximum electron transfer capacity (ETS) of phosphorylation respiration.

### Histological analysis

The excised liver tissue underwent Hematoxylin and Eosin (H&E) staining for histopathological analysis. Briefly, the freshly harvested liver was fixed in 4% paraformaldehyde, embedded in paraffin, sectioned into 4 μm-thick slices, and stained with H&E using established methods (Gao et al., 2020). For Oil Red O staining, Frozen liver was fixed, dehydrated, and cut into 8 μm-thick sections. Tissue sections were stained with saturated oil red O staining solution for 5 min, then washed briefly in isopropanol to keep the sections moist. Afterwards, glycerol aqueous solution was added to seal the sections and observed. The stained sections were then evaluated using the Nonalcoholic Steatohepatitis Activity Score system, which assesses liver steatosis, inflammation, and ballooning (Kleiner et al., 2005).

### Biochemical parameters

Fasting for 12 h is required before blood collection. Commercial kits for measuring the levels of alanine aminotransferase (ALT), aspartate aminotransferase (AST), total cholesterol (TC), and triglycerides (TG) in serum were obtained from Mindray Bio-Medical Electronics Co., Ltd. (Shenzhen, China). Kits for HDL-C and LDL-C were also sourced from Mindray Bio-Medical Electronics Co., Ltd (Shenzhen, China). The aforementioned biochemical indicators in the serum were determined on an automatic biochemical analyzer (Mindray Automatic Biochemical Analyzer, Shenzhen, China) according to the manufacturer’s instructions.

### SPR analysis

The binding affinity between Silibinin A or Silibinin B and the proteins SDHA, SDHB, SDHC, and SDHD immobilized on a CM5 sensor chip was detected using the Biacore T200 system at 25°C (Biacore, GE Healthcare, Sweden). In brief, after testing the buffer suitability for pH, the proteins SDHA, SDHB, SDHC, and SDHD were immobilized on an activated carboxymethylated 5 (CM5) sensor chip using amine coupling. Silibinin A or Silibinin B at gradient concentrations were then injected at a flow rate of 30 μL/min in the running buffer [PBS with 0.05% (v/v) Tween 20% and 5% (v/v) DMSO]. The interaction was monitored, and the results were analyzed using the Biacore evaluation software (T200 version 2.0). The data were fitted to a 1:1 Langmuir binding model, and the equilibrium constant (KD) was calculated (Fu et al., 2021).

### Molecular docking

The X-ray crystal structures of SDHA were obtained from the Protein Data Bank (PDB code: 6VAX), while the crystal structure of SDHB was obtained from the Protein Data Bank (PDB code: 8GS8). The 3D structures of silibinin A and silibinin B were downloaded from the PubChem databases (https://pubchem.ncbi.nlm.nih.gov/). Molecular docking was performed using AutoDock Vina. Docked poses were analyzed using PyMOL (Free and open-source version). According to the research instructions of Wang et al. (2024).

### Statistical analysis

All data were analyzed using SPSS software and expressed as mean ± SEM. Differences between two groups were assessed using Student’s t-test. For multiple comparisons, one-way analysis of variance (ANOVA) was applied, followed by Tukey’s *post hoc* test. Statistical significance was considered at a *p*-value less than 0.05. The statistical methods for each experiment are indicated in the figure legends. Animals with similar baseline values were randomly assigned to each group using simple random sampling. Data for animal studies were collected in a blinded manner. All *in vitro* and *in vivo* experiments were independently repeated at least three times.

## Results

### Silibinin improves PM2.5-induced triglyceride accumulation in the liver tissue of C57BL/6J mice

**FIGURE 1:**
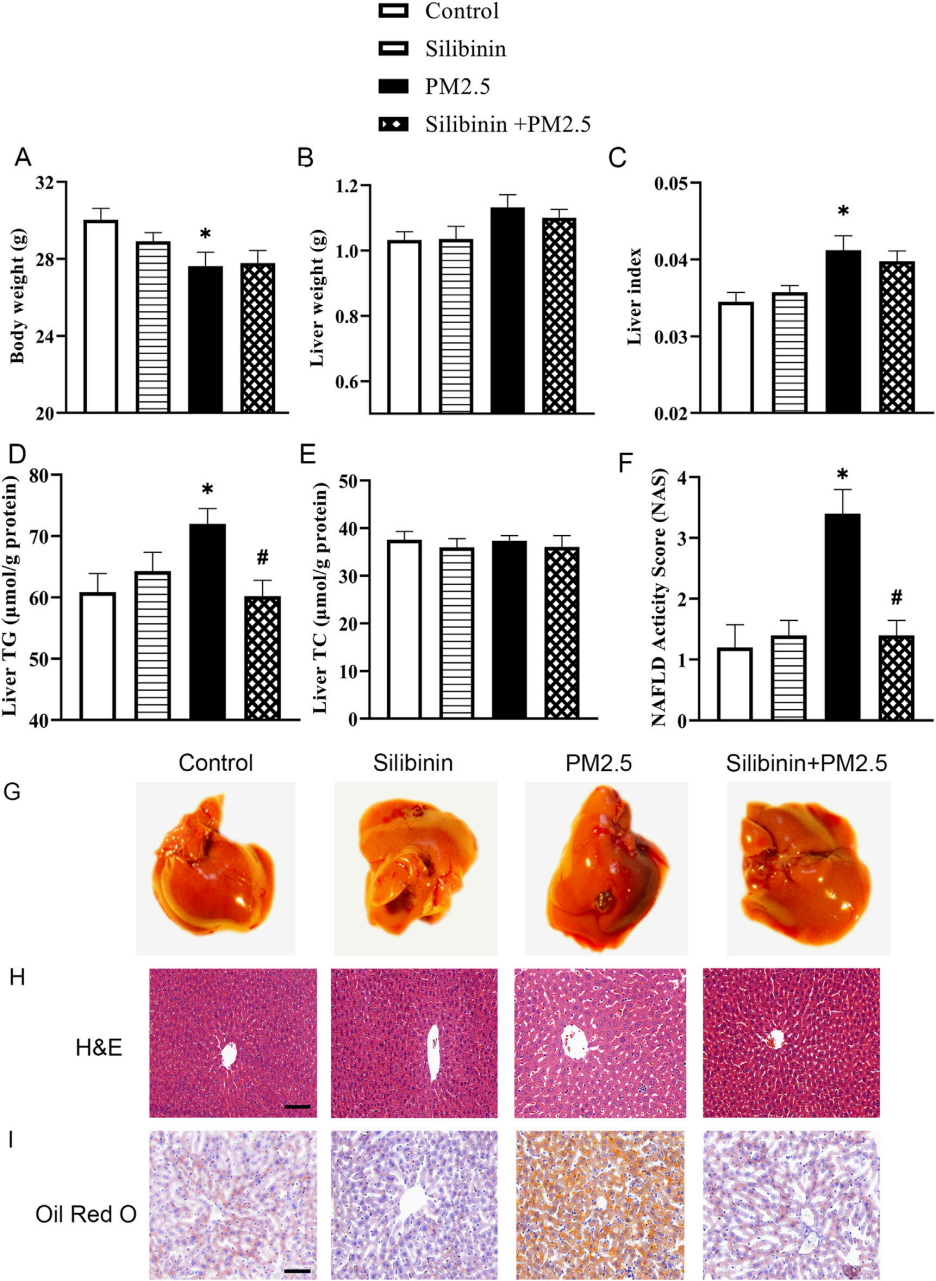
Silibinin improves PM2.5-induced triglyceride accumulation in the liver tissue of C57BL/6J mice. **(A–C)** Silibinin treatment on mice exposed to PM2.5: evaluating changes in body weight **(A)**, liver weight **(B)**, and liver index **(C)**. n = 8 replicates. **(D,E)** Silibinin treatment on mice exposed to PM2.5: evaluating concentrations of intrahepatic triglycerides (TG) **(D)** and total cholesterol (TC) **(E)**. n = 6 replicates. **(F)** NAFLD activity scores (NAS). n = 5 replicates. **(G–I)** The images of hepatic macroscopic overview **(G)**, hematoxylin and eosin**(H&E)** staining **(H)** and Oil Red O staining **(I)** from different groups, Scale bar = 50 μm. n = 4 replicates. The data mentioned earlier were presented as means ± SEMs and subjected to one-way ANOVA for analysis.*p < 0.05 vs. NC. ^#^ *p* < 0.05 vs PM2.5.

In order to directly assess the protective effect of silibinin on the liver of mice following intratracheal instillation of PM2.5, we conducted intratracheal instillation of PM2.5 followed by oral administration of silibinin. The results showed that PM2.5 exposure resulted in a significant decrease in body weight (Figure 1A), an significant increase in liver index (Figure 1C), and a significant elevation in triglycerides (Figure 1D). Importantly, the silibinin was able to reverse the accumulation of intrahepatic triglycerides induced by PM2.5 in mice (Figure 1D). The liver weight and total cholesterol levels showed no significant differences among the groups (Figures 1B, E).

These observations, further supported by H&E and Oil Red O staining, confirmed the accumulation of lipids in the liver due to PM2.5 exposure (Figures 1G–I). To assess the severity of Non-Alcoholic Fatty Liver Disease (NAFLD). We calculated the NAFLD activity score. The results indicated that the PM2.5 group had a significantly higher score compared to the Control group (Figure 1F), which improved following treatment with silibinin. The serum biochemical results showed that PM2.5 significantly increased the levels of AST, LDL-C, and TG in the serum, which could be significantly suppressed by silibinin treatment, except for TG. (Supplementary Figures S1D, F, H). There were no significant changes in the levels of ALT, HDL-C, and TC in the serum among the groups (Supplementary Figures S1C, E, G).

In addition, qPCR analysis was conducted to evaluate the levels of inflammation-related genes [Interleukin-1α (*IL-1*α), Interleukin-1β (*IL-1*β), NOD-Like Receptor Family, Pyrin Domain Containing 3 (*NLRP3*), Tumor Necrosis Factor-alpha (*TNF-*α), and Interleukin-8 (*IL-8*)] in liver tissue stimulated by PM2.5. The results indicated that under PM2.5 exposure, the expression of inflammation-related genes *IL-1*β and *TNF-*α increased significantly, which was suppressed by silibinin treatment (Supplementary Figure S2A). Similarly, regarding antioxidant-related genes [Nuclear factor erythroid 2-related factor 2 (*Nrf2*), Heme oxygenase-1 (*HO-1*), NAD(P)H Quinone Oxidoreductase 1 (*NQO-1*), Glutamate-cysteine ligase catalytic subunit (*GCLC*) and Glutathione S-transferase (*GST*)], PM2.5 stimulation notably decreased the expression of *GCLC*, while silibinin treatment reversed the reduced expression of *GCLC* (Supplementary Figure S2B). Interestingly, compared to the control group, the silibinin group significantly increased the expression of antioxidant-related genes (*Nrf2, HO-1, GCLC*, and *GST*) (Supplementary Figure S2B).

### Exposure to PM2.5 significantly induces the accumulation of triglycerides within hep G2 cells

**FIGURE 2:**
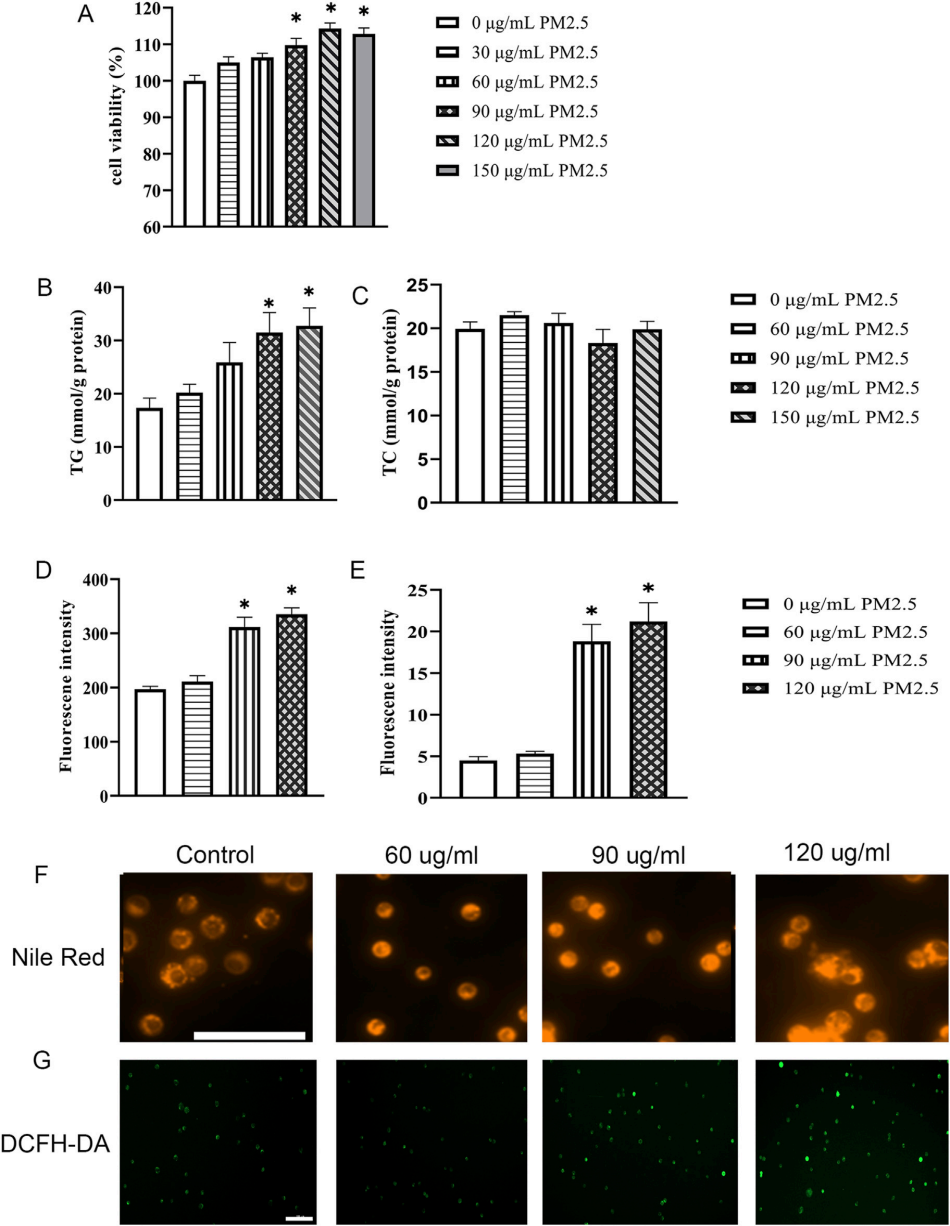
Exposure to PM2.5 significantly induces the accumulation of triglycerides within Hep G2 cells. **(A)** CCK-8 assays were performed to evaluate the activity levels of Hep G2 cells after 72 h of cultivation at various PM2.5 concentrations (0–150 μg/mL). n = 6 replicates. **(B,C)** Cellular TG content **(B)** and TC content **(C)** were determined using the GPO Trinder enzymatic reaction after exposing the cells to varying concentrations of PM2.5 (0–150 μg/mL) for 72 h n = 3 replicates **(D,E)** Quantitative measurements of Nile Red **(D)** and DCFH-DA **(E)**. n = 3 replicates. **(F–G)** Fluorescence images show lipid droplets stained with Nile Red **(F)** and reactive oxygen species (ROS) labeled with DCFH-DA **(G)** in cells exposed to different concentrations of PM2.5 (0–120 μg/mL) for 72 h. Scale bar = 100 μm. The data mentioned earlier were presented as means ± SEMs and subjected to one-way ANOVA for analysis. *p < 0.05 vs 0 μg/mL PM2.5.

After 24 h of ultrasonic treatment, the PM2.5 aggregate particles exhibit a uniform distribution, and aggregated particles cannot be detected (Supplementary Figure S3). Subsequently, the impact of PM2.5 on Hep G2 cells was assessed. Compared to 0 μg/mL PM2.5, cell viability increased in a concentration-dependent manner after 72 h of PM2.5 stimulation. 90, 120, and 150 μg/mL PM2.5 significantly increased cell viability (Figure 2A). After exposure to different concentrations of PM2.5 (0–150 μg/mL) for 72 h, Hep G2 cell triglyceride (TG) (Figure 2B) and total cholesterol (TC) (Figure 2C) contents were determined using the GPO Trinder enzymatic method. The results showed a significant increase in intracellular triglyceride levels at PM2.5 exposures of 120 μg/mL and 150 μg/mL (Figure 2B), while no impact on total cholesterol was observed (Figure 2C). To visualize the extent of lipid droplet accumulation in Hep G2 cells, Nile red dye (Figure 2F) was used to detect the distribution and quantity of lipid droplets. The results demonstrated a significant increase in lipid droplets in Hep G2 cells after exposure to 90 and 120 μg/mL PM2.5 (Figure 2D). Furthermore, we used the DCFH-DA fluorescent probe to detect ROS levels in treated cells (Figure 2G) and demonstrated that ROS levels significantly increased at PM2.5 concentrations of 90 and 120 μg/mL (Figure 2E). Following previous relevant studies (Ding et al., 2019; Wang et al., 2020), the appropriate working concentrations of PM2.5 (120 μg/mL) were selected for subsequent experiments.

### Silibinin inhibits PM2.5-induced triglyceride accumulation in hep G2 cells

**FIGURE 3:**
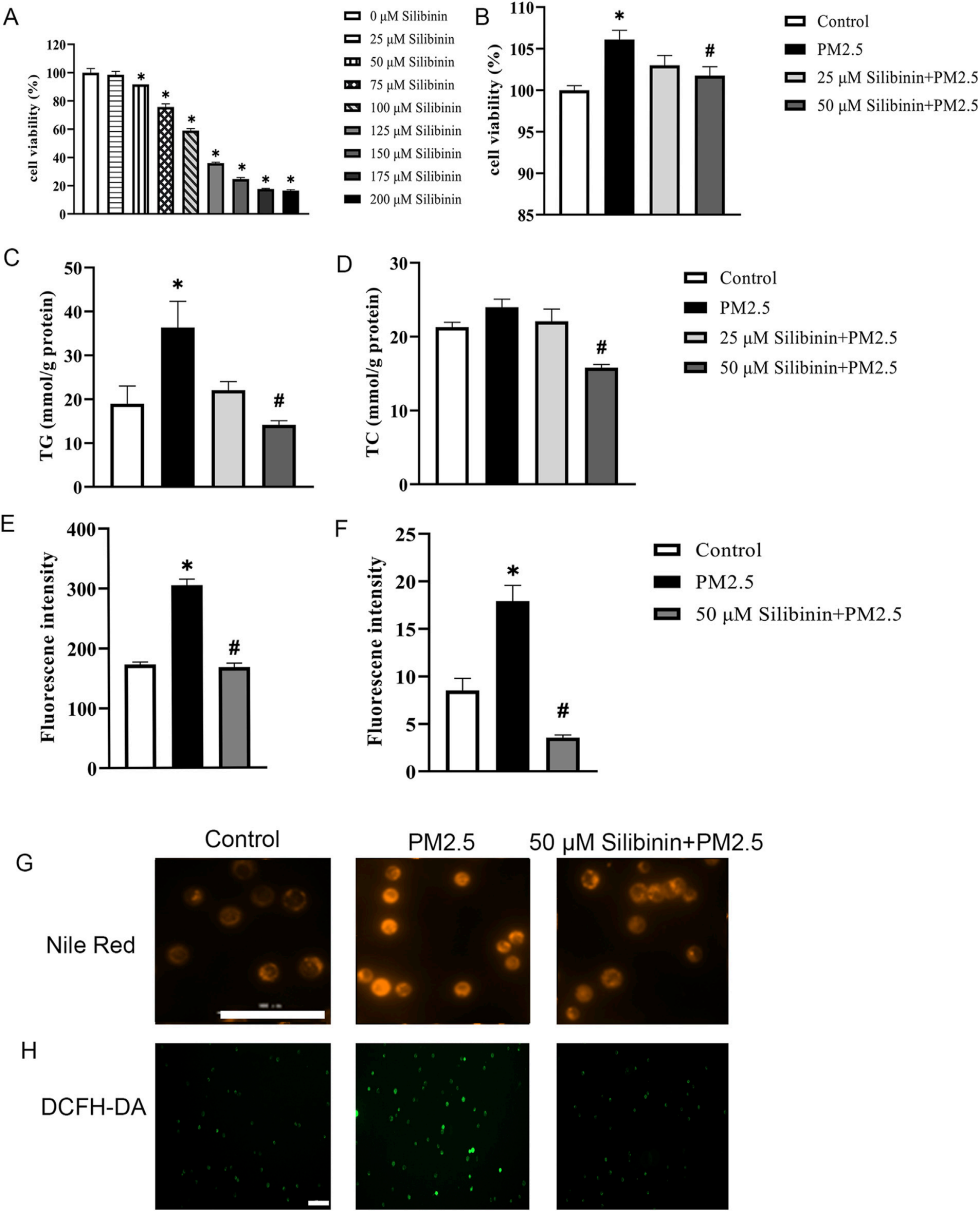
Silibinin inhibits PM2.5-induced triglyceride accumulation in Hep G2 cells. **(A,B)** CCK-8 assays were performed to evaluate the activity levels of Hep G2 cells after 72 h of cultivation at various silibinin concentrations (0–200 μM) **(A)** and co-treated with 120 μg/mL PM2.5 and silibinin **(B)**. n = 6 replicates. **(C,D)** Cellular TG content **(C)** and TC content **(D)** were determined using the GPO Trinder enzymatic reaction after exposing the cells to PM2.5 (120 μg/mL) and co-treated with 120 μg/mL PM2.5 and silibinin (25,50 μM) **(D)**. n = 3 replicates. **(E,F)** Quantitative measurements of Nile Red **(E)** and DCFH-DA **(F)**. n = 3 replicates. **(G,H)** Fluorescence images show lipid droplets stained with Nile Red. bar = 100 μm **(G)** And reactive oxygen species (ROS) labeled with DCFH-DA **(H)** in cells exposed to PM2.5 and co-treated with 120 μg/mL PM2.5 and silibinin for 72 h. Scale bar = 100 μm. The data mentioned earlier were presented as means ± SEMs and subjected to one-way ANOVA for analysis. *p < 0.05 vs Control; #*p* < 0.05 vs PM2.5.

Next, we assessed the impact of silibinin on Hep G2 cells. CCK-8 assay revealed that, after 72 h of cultivation, cell viability significantly decreased with increasing concentrations of silibinin compared to 0 μM silibinin (Figure 3A). Within silibinin concentrations of 50 μM and below, Hep G2 cells maintained relatively high cell viability (cell viability ≥90%), hence 50 μM of silibinin was selected for subsequent experiments. Cell viability was significantly inhibited when silibinin was added simultaneously with PM2.5 stimulation (Figure 3B).

Using the GPO Trinder enzymatic method, intracellular triglyceride (TG) and total cholesterol (TC) contents were determined. The results indicated that silibinin significantly inhibited the increase in intracellular triglycerides induced by PM2.5 (Figure 3C). Furthermore, compared to the PM2.5 group, silibinin treatment significantly reduced intracellular total cholesterol (Figure 3D). Additionally, Nile red dye was used to detect the distribution and quantity of lipid droplets (Figure 3G). The results showed that intracellular lipid droplets significantly increased after PM2.5 exposure, while silibinin treatment significantly inhibited their accumulation (Figure 3E). To examine whether silibinin could suppress the increase in ROS under PM2.5 stimulation, the results show that PM2.5-induced ROS production significantly increased, and silibinin inhibited the effects (Figure 3H). These results suggest that silibinin may improve mitochondrial dysfunction by reducing ROS production (Figure 3F). These results suggest that silibinin inhibits PM2.5-induced triglyceride accumulation in Hep G2 cells.

### Silibinin affects oxidative stress and antioxidant gene expression induced by PM2.5 in Hep G2 cells

**FIGURE 4:**
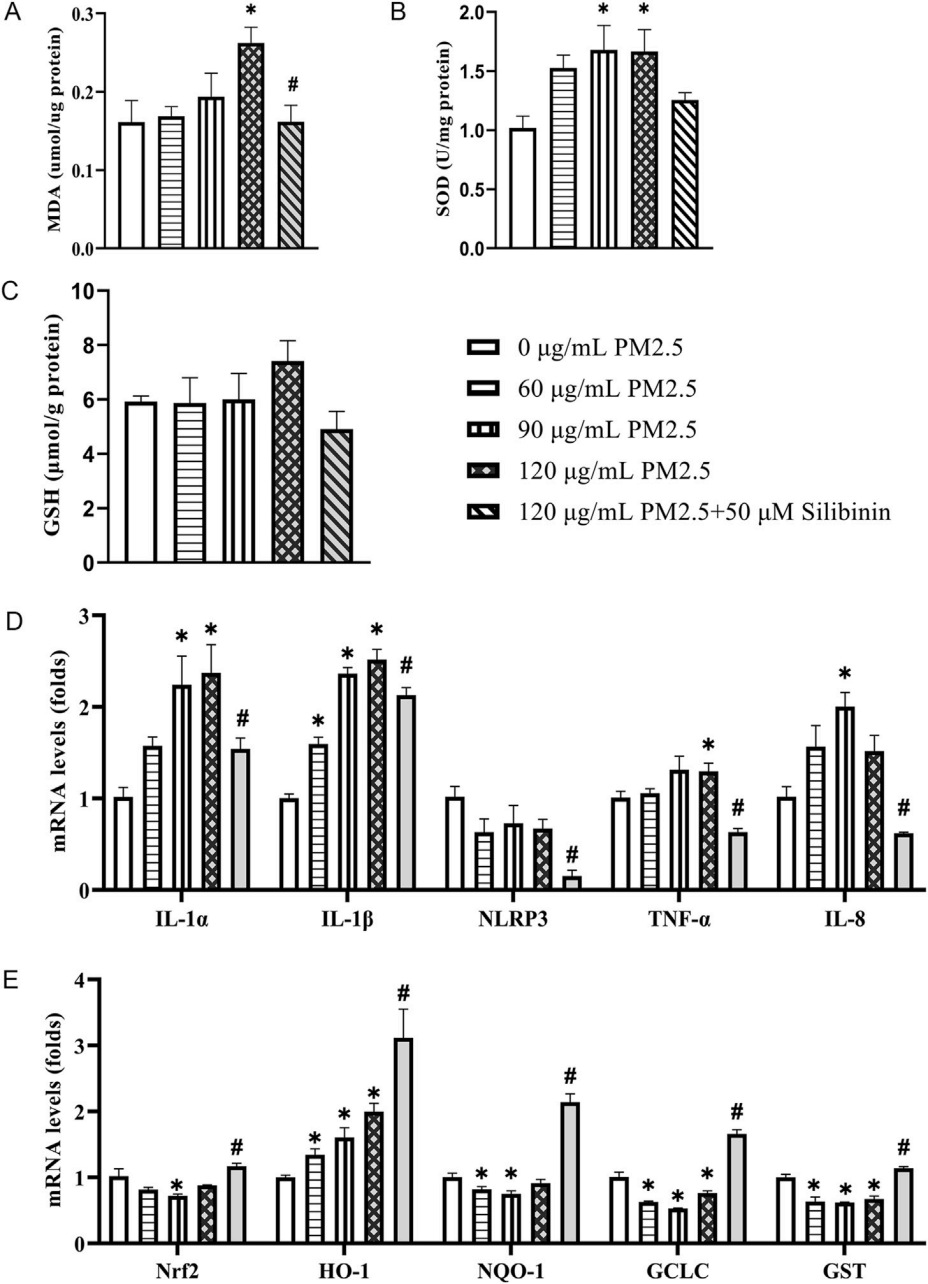
Silibinin inhibits the increase in oxidative stress and upregulation of inflammatory gene expression induced by PM2.5 in Hep G2 cells. **(A–C)** Determine the activity levels or concentrations of MDA **(A)**, SOD **(B)** and GSH **(C)** in Hep G2 cells treated with varying concentrations of PM2.5 suspension, and co-treated with 120 μg/mL PM2.5 and silibinin, using commercially available assay kits. n = 4 replicates. **(D)** qPCR analyses of pro-inflammatory factors *IL-1*α, *IL-1*β, *NLRP3, TNF-*α and *IL-8* levels in Hep G2 cells. n = 4 replicates **(E)** qPCR analyses of anti-oxidants *Nrf2, HO-1, NQO-1, GCLC* and *GST* levels in Hep G2 cells among the five groups. n = 4 replicates. The data mentioned earlier were presented as means ± SEMs and subjected to Student’s t-test for analysis. *p < 0.05 vs 0 μg/mL PM2.5; ^#^ *p* < 0.05 vs 120 μg/mL PM2.5.

We used commercial assay kits to assess the concentrations or activity levels of GSH, MDA, and SOD in Hep G2 cells treated with different concentrations of PM2.5 (0–150 μg/mL) and treated with PM2.5 and silibinin for 72 h. The results showed that compared to 0 μg/mL PM2.5, 120 μg/mL PM2.5 significantly increased MDA content, which was inhibited by silibinin treatment (Figure 4A). SOD activity significantly increased at 90 μg/mL and 120 μg/mL PM2.5 (Figure 4B). There were no significant differences in GSH levels between the groups (Figure 4C).

Furthermore, qPCR analysis was performed to assess the levels of inflammation-related genes (*IL-1*α, *IL-1*β, *NLRP3, TNF-*α, and *IL-8*) in Hep G2 cells stimulated with PM2.5 at different concentrations. The results showed an increase in the expression of inflammation-related genes under PM2.5 stimulation at various concentrations (Figure 4D). Specifically, 120 μg/mL PM2.5 significantly increased the expression of *IL-1*α, *IL-1*β and *TNF-*α, which was suppressed by silibinin treatment. Similarly, for antioxidant-related genes, stimulation with PM2.5 (120 μg/mL) significantly decreased the expression of *GCLC*, and *GST*, which was reversed by silibinin treatment (Figure 4E).

Nicotinamide Adenine Dinucleotide, (NAD+) has been reported as essential in redox reactions, and a decrease in its levels can disrupt various physiological processes (Chini et al., 2024), including energy metabolism, redox homeostasis, and cell signal transduction. We measured the levels of NAD+/NADH and NADP+/NADPH in the three groups using commercial assay kits. The results showed that compared to the Control group, PM2.5 treatment significantly reduced NAD total (Supplementary Figure S4A), NAD+ (Supplementary Figure S4B), and Nicotinamide Adenine Dinucleotide, reduced form (NADH) (Supplementary Figure S4C) levels, as well as significantly decreased total Nicotinamide Adenine Dinucleotide Phosphate (NADP total) (Supplementary Figure S4E) and Nicotinamide Adenine Dinucleotide Phosphate, reduced form (NADPH) (Supplementary Figure S4G) levels. However, silibinin treatment significantly inhibited the decrease in total NAD (Supplementary Figure S4A), total NADP (Supplementary Figure S4E), NAD+ (Supplementary Figure S4B) and NADPH (Supplementary Figure S4G) levels induced by PM2.5. Additionally, there were no significant differences in the ratios of NAD+/NADH and NADP+/NADPH among the three groups (Supplementary Figures S4D, H).

**FIGURE 5:**
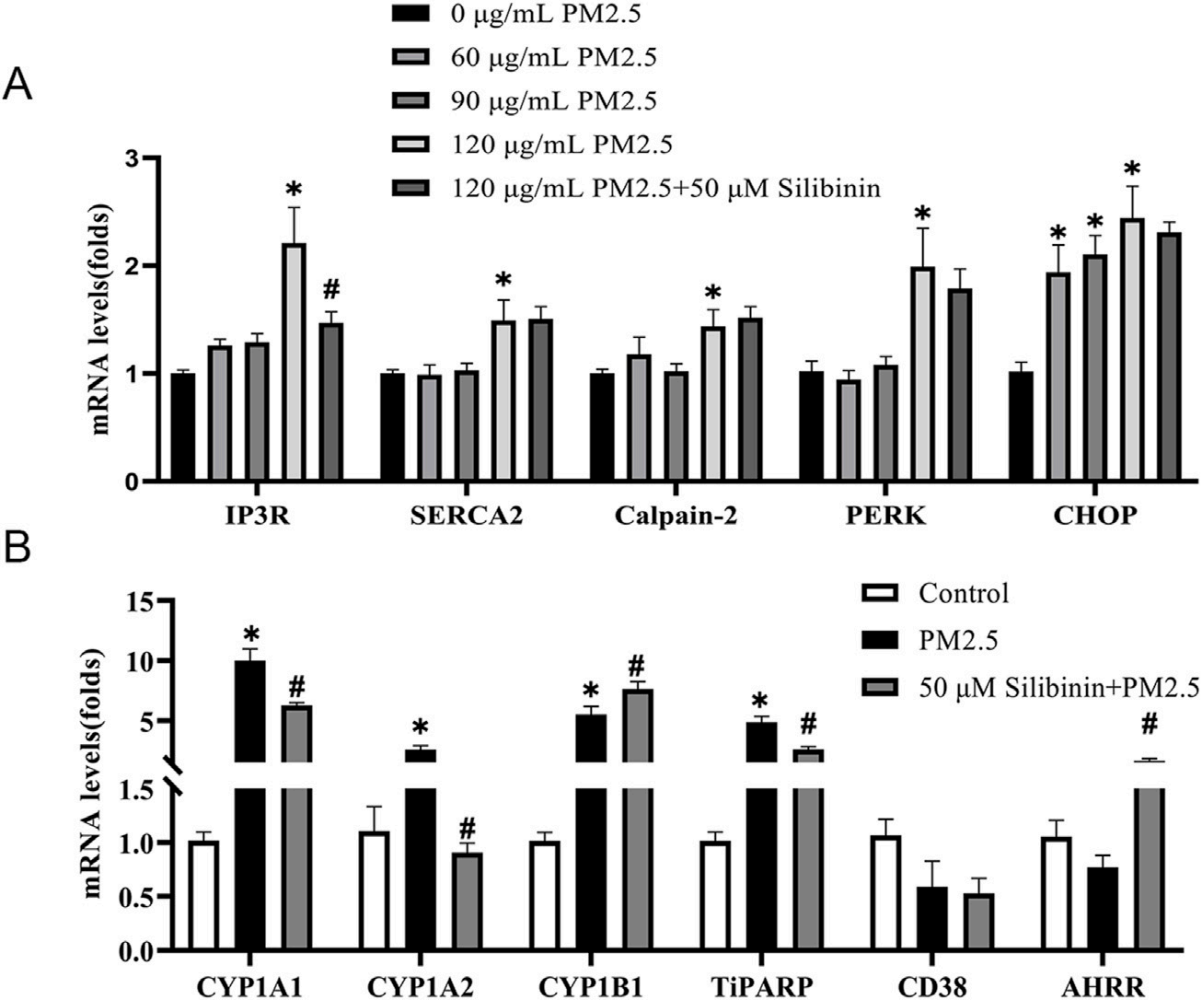
Real-time PCR validation of the regulation of calcium-related genes and aromatic hydrocarbon receptor downstream genes across different groups in Hep G2 cells. **(A)** qPCR analyses of calcium-related genes *IP3R, SERCA2, Calpain-2, PERK* and *CHOP* levels in Hep G2 cells among the five groups. n = 4 replicates **(B)** qPCR analyses of aromatic hydrocarbon receptor downstream genes *CYP1A1, CYP1A2, CYP1B1, TiPARP, CD38*, and *AHRR* levels in Hep G2 cells among the three groups. n = 4 replicates. The data mentioned earlier were presented as means ± SEMs and subjected to Student’s t-test for analysis. *p < 0.05 vs Control; ^#^ *p* < 0.05 vs PM2.5.

PM2.5 comprises various components that, upon contact with tissues and cells, can trigger the overexpression of genes related to the downstream effects of aromatic hydrocarbons (Liu et al., 2024). Such upregulation of gene expression may alter the redox state within hepatocytes by increasing intracellular ROS and depleting NAD+, thereby promoting the onset and progression of non-alcoholic fatty liver disease. Furthermore, the release of calcium ions is closely associated with ROS production. Our results show that PM2.5 exposure (120 μg/mL) significantly elevates the expression of Inositol 1,4,5-trisphosphate receptor (*IP3R*), Sarcoplasmic/Endoplasmic Reticulum Calcium ATPase (*SERCA2*), *Calpain-2*, Protein Kinase RNA-like Endoplasmic Reticulum Kinase (*PERK*), and C/EBP-homologous protein (*CHOP*), while silibinin treatment can suppress the increased expression of IP3R (Figure 5A). There data suggest that PM2.5 triggers oxidative stress in a dose-dependent manner in Hep G2 cell. Our research findings indicate that, compared to the control group, PM2.5 exposure leads to a significant increase in the expression of Cytochrome P450 1A1 (*CYP1A1*), Cytochrome P450 1A2 (*CYP1A2*), Cytochrome P450 1B1 (*CYP1B1*), and TCDD-inducible poly [ADP-ribose] polymerase (*TiPARP*), which are downstream genes of the aromatic hydrocarbon receptor (AHR) (Figure 5B). However, this trend can be reversed with silibinin treatment.

### Quantitative proteomic study conducted on liver tissue

**FIGURE 6:**
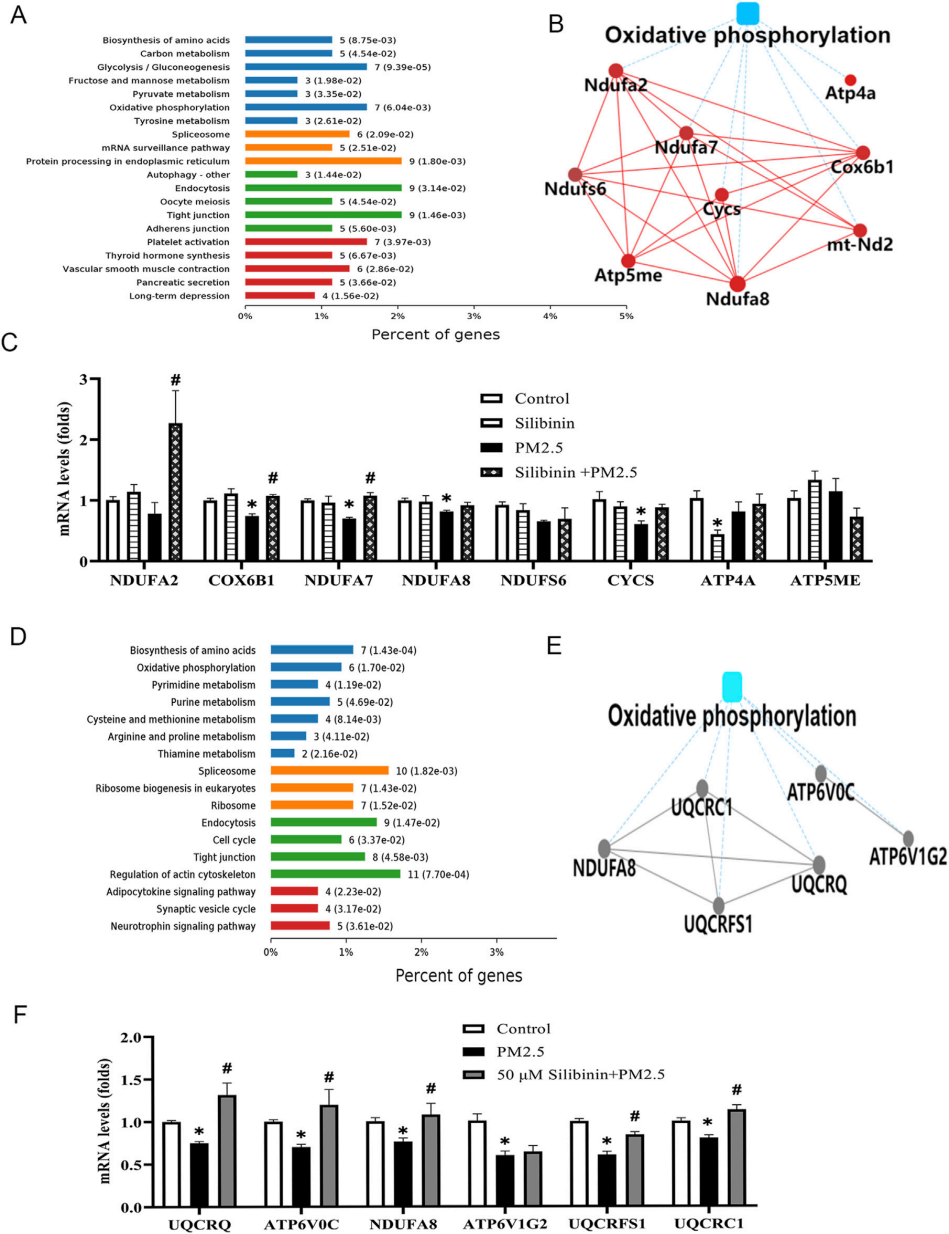
Quantitative proteomic study conducted on liver tissue and Hep G2 cells among the groups. **(A)** The KEGG pathway enrichment results indicate the involvement of cellular pathways related to oxidative phosphorylation in liver tissue **(B)** The image illustrates the Protein-Protein Interaction analysis using String Database and Cytoscape software in liver tissue. **(C)** Quantitative PCR (qPCR) was employed to analyze the levels of key genes (*NDUFA2, COX6B1, NDUFA7, NDUFA8, NDUFS6, CYCS, ATP4A*, and *ATP5ME*) associated with the oxidative phosphorylation in liver tissue witnin the four groups. **(D)** The KEGG pathway enrichment results indicate the involvement of cellular pathways related to oxidative phosphorylation in Hep G2 cells **(E)** The image illustrates the Protein-Protein Interaction analysis using String Database and Cytoscape software in Hep G2 cells. **(F)** Quantitative PCR (qPCR) was employed to analyze the levels of key genes (*UQCRQ, ATP6V0C, NDUFA8, ATP6V1G2, UQCRFS1*, and *UQCRC1*) associated with the oxidative phosphorylation pathway in Hep G2 cells witnin the three groups. n = 4 replicates. The data mentioned earlier were presented as means ± SEMs and subjected to Student’s t-test for analysis.*p < 0.05 vs. Control; #*p* < 0.05 vs PM2.5.

Quantitative proteomics were employed to detect changes in protein expression in the study. The results indicated that these proteins were associated with oxidative phosphorylation (Figure 6A). Additionally, protein-protein interaction analysis was performed using the String database and Cytoscape software (Figure 6B). This analysis specifically focused on proteins related to oxidative phosphorylation within the metabolic pathway. Subsequently, qPCR analysis was carried out on the differentially expressed proteins involved in oxidative phosphorylation and other related pathways. The qPCR results (Figure 6C) revealed that the expression levels of key genes (Cytochrome c Oxidase Subunit 6B1 (*COX6B1*), Mitochondrial Complex I Subunit 7 (*NDUFA7*) Mitochondrial Complex I Subunit 8 (*NDUFA8*) and Cytochrome c (*CYCS*)) in the oxidative phosphorylation pathway significantly decreased after PM2.5 stimulation. However, silibinin treatment significantly attenuated the reduction in the expression levels of all key genes except *NDUFA8* and *CYCS*.

### Quantitative proteomic study conducted on human-derived hep G2 cells

Quantitative proteomics were employed to detect changes in protein expression in the study. The results indicated that these proteins were associated with oxidative phosphorylation (Figure 6D). To further explore the molecular mechanisms underlying the lipid-lowering effect of silibinin, protein-protein interaction analysis was also performed using the String database and Cytoscape software (Figure 6E). The analysis focused on proteins related to oxidative phosphorylation in the metabolic pathway. Finally, qPCR analysis was conducted on the differentially expressed proteins in the oxidative phosphorylation pathway, including *UQCRQ, ATP6V0C, NDUFA8, ATP6V1G2, UQCRFS1*, and *UQCRC1*. The qPCR results (Figure 6F) showed that the key gene expression levels in the oxidative phosphorylation pathway of Hep G2 cells significantly decreased after PM2.5 stimulation. However, silibinin treatment significantly inhibited the decrease in the expression levels of all key genes except *ATP6V1G2*.

### Exposure to PM2.5 resulted in a decline in both complex I and II function of mitochondrial complexes

**FIGURE 7:**
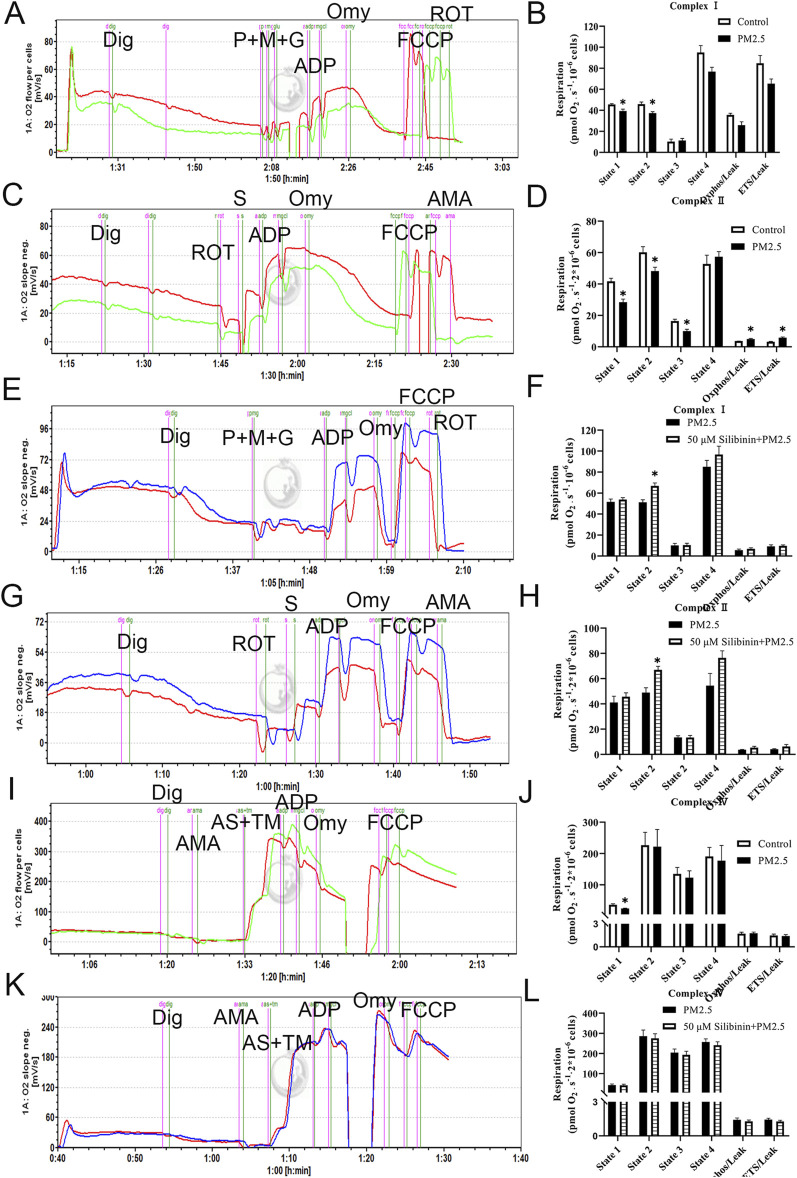
Exposure to PM2.5 resulted in a decline in the function of mitochondrial complexes I and II, but silibinin reversed this trend. **(A,B)** The representative results (left) and statistical data (right) of complex I-related respiration in Hep G2 liver cells, treated with Digoxin (Dig) in the presence of PM2.5, are presented. n = 3 replicates. **(C,D)** The representative (left) and statistical (right) results of complex II-related respiration in Hep G2 liver cells, treated with Digoxin (Dig) in the presence of PM2.5, are shown. n = 4 replicates. **(E,F)** Complex I respiratory function in Hep G2 liver cells (permeabilized with Digoxin after PM2.5 exposure) under treatment with Silibinin illustrated by representative results (left) and supported by statistical findings (right). n = 4 replicates. **(G,H)** Complex II respiratory function in Hep G2 liver cells (permeabilized with Digoxin after PM2.5 exposure) under the influence of silibinin treatment is elucidated through representative (left) and statistical (right) results. n = 5 replicates. **(I,J)** The representative (left) and statistical (right) results of complex IV-related respiration in Hep G2 liver cells, treated with Digoxin (Dig) in the presence of PM2.5, are depicted. n = 4 replicates. **(K,L)** Complex IV respiratory function in Hep G2 liver cells (permeabilized with Digoxin after PM2.5 exposure) treated with silibinin is illustrated by representative (left) and statistical (right) results. n = 4 replicates. The data mentioned earlier were presented as means ± SEMs and subjected to Student’s t-test for analysis *p < 0.05 vs. Control or PM2.5.

Recent studies have shown that the transition from simple steatosis to definite non-alcoholic steatohepatitis (NASH) is closely associated with a decline in mitochondrial function. In addition, the results from the previous proteomic experiment indicated that the lipid-lowering mechanism of silibinin is closely associated with the oxidative phosphorylation pathway. Additionally, the qPCR experiments confirmed these results. The results related to NAD+/NADH and NADP+/NADPH also confirmed their correlation with mitochondrial function. To further determine whether the molecular mechanism underlying the lipid-lowering effect of silibinin involves enhancing the respiratory capacity of various electron transport chain complexes on permeabilized Hep G2 cells, we assessed the respiratory capacity. Interestingly, compared to the Control group, significant inhibition of state two respiration induced by substrates of complex I (pyruvate, malate, and glutamate - P + M + G) (Figures 7A, B) and complex II (succinate - S) was observed in the presence of PM2.5 (Figures 7C, D). However, there was no impact on state two respiration induced by substrates of complex IV (ascorbate and TMPD - AS + TM) (Figures 7I, J). These results suggest that PM2.5 significantly impairs the oxidative phosphorylation function of mitochondrial complexes I and II.

### Silibinin inhibits the PM2.5-induced impairment of mitochondrial complex I and II functions

We assessed the specific respiration of mitochondrial complexes I, II, and IV. The results showed that, compared to the PM2.5 group, the silibinin group could reverse the state two respiration induced by substrates of complex I (pyruvate, malate, and glutamate - P + M + G) (Figures 7E, F) and complex II (succinate - S) (Figures 7G, H). However, there was no impact on state two respiration induced by substrates of complex IV (ascorbate and TMPD - AS + TM) (Figures 6K, L). These experimental results indicate that silibinin can inhibit the significant decrease in oxidative phosphorylation function of mitochondrial complexes I and II caused by PM2.5 After intervention with silibinin alone, the function of mitochondrial complex I decreased (Supplementary Figure S5A, B), while the function of mitochondrial complex II increased (Supplementary Figure S5C, D).

### Mitochondrial complex II (succinate dehydrogenase, SDH) is the direct target of silibinin *in vivo*

Currently, there is no direct evidence indicating that mitochondrial Complex II (SDH) is the direct target of silibinin *in vivo*. However, our results (Figure 7) have shown that silibinin can significantly enhance the activity of Complex II. To accurately assess the affinity of Silibinin A and Silibinin B towards target proteins within mitochondrial complex 2, we purchased commercial drugs for subsequent experiments. Silibinin A and Silibinin B, the two most crucial active compounds in silymarin, constitute approximately 60%–70% of its abundance and exist in two diastereomeric forms with a nearly equimolar ratio (Palit et al., 2021). However, there is limited literature on whether these two compounds differ in their pharmacokinetic properties and biological functions *in vivo*.

**FIGURE 8:**
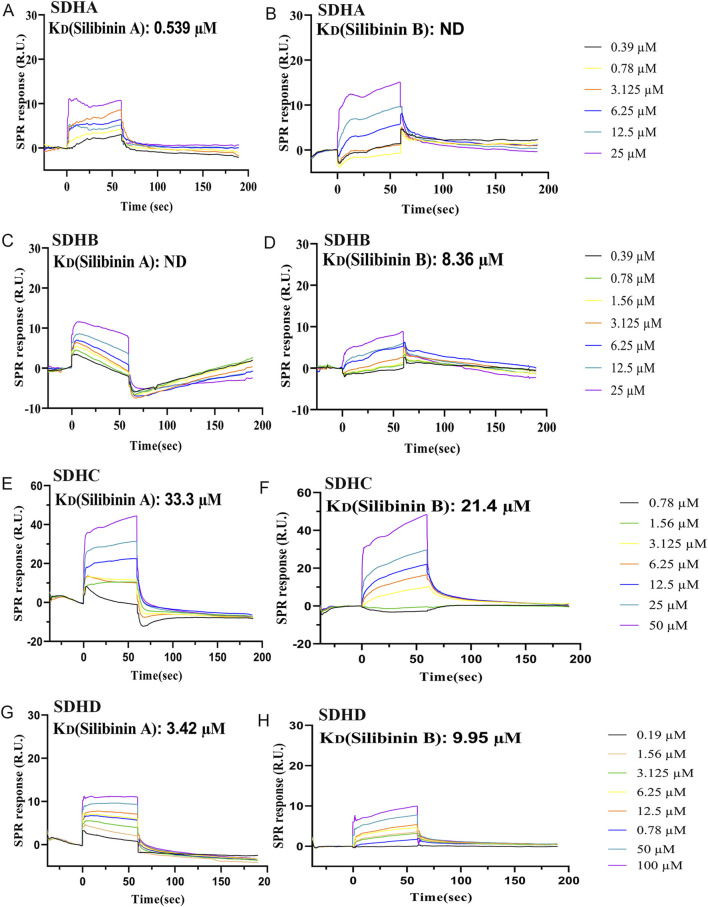
Characterization of the affinity between Silibinin A or Silibinin B and SDHA, SDHB, SDHC, and SDHD protein, which was immobilized on a CM5 sensor chip, based on the SPR assay. **(A,B)** SPR sensorgrams of the binding of Silibinin A **(A)** or Silibinin B **(B)** to SDHA proteins captured on a CM5 chip. **(C,D)** SPR sensorgrams of the binding of Silibinin A **(C)** or Silibinin B **(D)** to SDHB proteins captured on a CM5 chip. **(E,F)** SPR sensorgrams of the binding of Silibinin A **(E)** or Silibinin B **(F)** to SDHC proteins captured on a CM5 chip. **(G,H)** SPR sensorgrams of the binding of Silibinin A **(G)** or Silibinin B **(H)** to SDHD proteins captured on a CM5 chip.

To analyze the specific interactions between Silibinin A, Silibinin B, and the various subunits of mitochondrial complex 2, we subsequently employed surface plasmon resonance (SPR) as a method to measure their affinity. Our findings revealed that Silibinin A bound to SDHA (Figure 8A), SDHC (Figure 8E), and SDHD (Figure 8G) proteins immobilized on the CM5 chip. However, it did not bind to SDHB (Figure 8C), as evident from the detected response. Among these, SDHA exhibited the strongest binding affinity for Silibinin A, with a KD value of 0.539 μM. This suggests that Silibinin A may exert its effects by binding to the SDHA protein. Similarly, Silibinin B bound to SDHB (Figure 8D), SDHC (Figure 8F), and SDHD (Figure 8H) proteins immobilized on the CM5 chip, but did not bind to SDHA (Figure 8B). SDHB showed the strongest binding affinity for Silibinin B, with a KD value of 8.36 μM. This indicates that Silibinin B may function by binding to the SDHB protein.

Furthermore, our study revealed that while both Silibinin A and Silibinin B can bind to three subunits of mitochondrial complex 2, they differ in their affinity towards each subunit. Silibinin A demonstrated strong affinity for SDHA and SDHD proteins, with KD values of 0.539 μM and 3.42 μM, respectively. On the other hand, Silibinin B showed strong affinity for SDHB and SDHD proteins, with KD values of 8.36 μM and 9.95 μM, respectively. These differences could be attributed to their enantiomeric structures.

**FIGURE 9:**
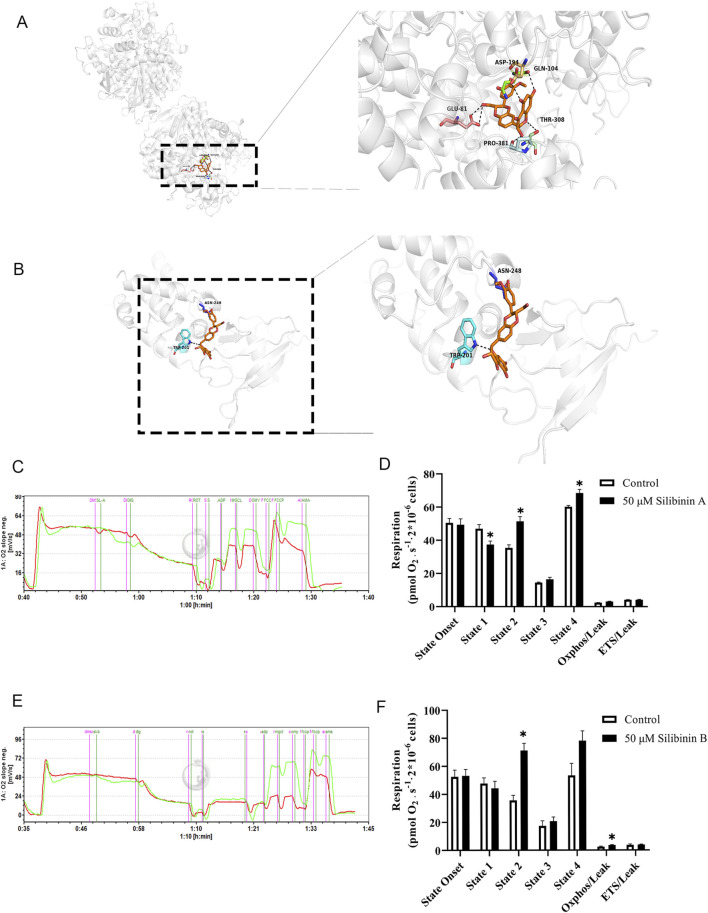
Characterization of Silibinin A or Silibinin B as a succinate dehydrogenase complex, subunit A or B activator. **(A)** Molecular modeling of the Silibinin A -succinate dehydrogenase complex, subunit A. **(B)** Molecular modeling of the Silibinin B **-**succinate dehydrogenase complex, subunit B. Key residues of the succinate dehydrogenase complex, subunit A or B were shown as sticks. Hydrogen bonds are shown as dashed. **(C,D)** Complex II respiratory function in Hep G2 liver cells (permeabilized with Digoxin) under the influence of Silibinin A treatment is elucidated through representative (left) and statistical (right)) results. n = 3 replicates. **(E,F)** Complex II respiratory function in Hep G2 liver cells (permeabilized with Digoxin) under the influence of Silibinin B treatment is elucidated through representative (left) and statistical (right) results. n = 7 replicates.

Next, we utilized molecular docking software to explore the binding sites of silibinin on mitochondrial complex II. As shown in (Figure 9A), silibinin A forms hydrogen bonds with the amino acids at the ASP (194), GLN (104), THR (308), PRO (381), and GLU (81) sites of SDHA. Meanwhile, silibinin B forms hydrogen bonds with the amino acids at the ASN (248) and TRP (201) sites of SDHB (Figure 9B). Taken together, these data suggest that silibinin A and B are effective agonists of mitochondrial complex II. Silibinin A and B, as non-superimposable diastereoisomers, may exhibit different pharmacological activities due to their distinct chemical spatial structures. This difference could lead to binding at different positions on amino acids within pockets on mitochondrial complexes. It is worth noting that silibinin A and silibinin B significantly increase the function of complex II within the cell (Figures 9C–F). These results suggest that mitochondrial complex II (succinate dehydrogenase, SDH) is the direct target of silibinin.

## Discussion

PM2.5 refers to particulate matter with a diameter of less than 2.5 µm (Hou et al., 2024), including carbon black, metals, nitrates, polycyclic aromatic hydrocarbons, and vehicle exhaust particles, among others. Particles with a diameter of less than 2.5 µm can be directly inhaled into the lungs and enter the bloodstream during respiration (Li et al., 2018), affecting the normal functions of organs such as the liver, kidneys, and even damaging the blood-brain barrier (Xu et al., 2016). This study found that exposure to PM2.5 leads to an accumulation of triglycerides, increased reactive oxygen species (ROS) production, elevated expression of inflammatory factors, decreased expression of antioxidant factors, and increased expression of downstream genes of aromatic hydrocarbon receptors. Silibinin can partially or fully reverse the aforementioned factors, thus protecting cells and the liver of animals from PM2.5-induced damage.

Firstly, PM2.5 has a significant impact on health. Previous studies have found that PM2.5 exposure leads to a significant decrease in body weight and a significant increase in liver index in mice (Zhu et al., 2023), which is consistent with our research results. Currently, many researchers generally agree that the negative effects of PM2.5 on human health are mainly realized through mechanisms such as oxidative stress, local inflammation, and systemic inflammation. These mechanisms interact with each other, exacerbating the harm of PM2.5 to human health. Inflammation and oxidative stress are considered key factors leading to metabolic-associated fatty liver disease. The occurrence of inflammation and oxidative stress is closely related to mitochondrial dysfunction (Zhu et al., 2023). The mitochondrial respiratory chain is the primary site of reactive oxygen species (ROS) generation within cells. Under normal conditions, a small amount of ROS is produced during electron transfer within the mitochondrial respiratory chain, serving as cellular signaling molecules. Our experimental results suggest that when mitochondrial respiratory function is impaired, there is an increase in electron leakage from the electron transport chain, leading to more electrons combining with oxygen to generate ROS. These findings align with observations made by other researchers (Chang et al., 2023), such as En-Ming Chang et al. and others, who have noted significant reductions in MMP, mitochondrial fragmentation, increased ROS levels (Wang et al., 2021), and mitochondrial dysfunction (Lin et al., 2022; You et al., 2024) in cells exposed to PM 2.5. Ning et al. (2021) further observed mitochondrial dysfunction in cells exposed to PM 2.5, characterized by excessive production of mitochondrial ROS, upregulation of superoxide dismutase 2 (SOD2) expression, decreased MMP, reduced oxygen consumption rate (OCR), decreased ATP levels, and increased intracellular Ca2+.

Furthermore, our study found that silibinin can inhibit the overexpression of *CYP1A1*, *CYP1A2*, and *TiPARP* mRNA in Hep G2 cells exposed to PM2.5. *CYP1A1, CYP1A2*, and *TiPARP* are genes downstream of the cellular aryl hydrocarbon receptor (AhR). The PM2.5 particles used in this study contained aromatic hydrocarbons, which, upon binding to AhR within cells, initiate the expression of downstream genes including *CYP1A1, CYP1A2, CYP1B1*, and *TiPARP*. During metabolism, these genes contribute to ROS production, which can attack DNA, lipids, and proteins, leading to liver damage (Chen et al., 2023) and triglyceride accumulation. Therefore, the protective effect of silibinin against PM2.5-induced liver injury may be attributed to its ability to inhibit the overexpression of *CYP1A1, CYP1A2*, and *TiPARP* mRNA. This suggests that silibinin could be a promising therapeutic agent for mitigating the adverse effects of PM2.5 exposure on hepatic health.

This study also found that succinate dehydrogenase (mitochondrial complex II) is a direct target of silibinin. Previous studies have shown that the decline in mitochondrial function is closely related to the progression from simple hepatic steatosis to non-alcoholic steatohepatitis (Staňková et al., 2020). Mitochondria are the site of oxidative metabolism in eukaryotes and play a crucial role in the final oxidation of carbohydrates, fats, and amino acids to release energy (Si et al., 2023). Our omics experiments indicate that the lipid-lowering mechanism of silibinin is closely related to the oxidative phosphorylation pathway, and this result was validated by qPCR. Similarly, Zhang et al. found (Bingqing Yang and Qi, 2024) in their study of non-alcoholic fatty liver disease induced by a high-fat diet that the expression levels of *NDUFB8* and *UQCRC2*, genes related to mitochondrial respiratory chain complexes, were significantly downregulated by the high-fat diet, and improved after drug treatment. Xue et al. (2024) established a mouse model of obesity induced by a high-fat diet using C57BL/6 mice and found that long-term feeding of a high-fat diet induced inflammation, a large number of lipid droplets in hepatocyte cytoplasm, significant swelling of mitochondria, rupture of cristae, and a decrease in ATP content, which could be reversed by drug treatment. Maseko et al. (2024) evaluated cellular energy metabolism, especially the impact on mitochondrial respiration, in Hep G2 cells treated with FFA *in vitro*. The results showed that basal respiration, maximal respiration, spare respiratory capacity, and ATP-linked respiration of Hep G2 cells treated with FFA were significantly decreased. Further studies have shown (Staňková et al., 2021) that in a NASH mouse model, complex II-driven respiration was significantly reduced, Sirtuin three expression was decreased, SDH subunit gene expression was reduced, and the level of the important signaling molecule liver succinate was increased. Overall, these results indicate that a decrease in mitochondrial respiration and an increase in ROS production are observed in metabolic-associated fatty liver disease. Consistent with the above results, our measurement of cellular energy metabolism and mitochondrial complex function revealed that PM2.5 led to a decrease in oxidative phosphorylation of mitochondrial complexes I and II, while silibinin inhibited the PM2.5-induced decrease in oxidative phosphorylation of mitochondrial complexes I and II. These results suggest that silibinin may protect the liver from damage by preserving mitochondrial complex function and reducing ROS generation.

Then, studies have shown (Scialò et al., 2017; Chavda and Lu, 2023) that enhancing the function of mitochondrial complex II promotes the transition from normal forward electron transport along the respiratory chain to reverse electron transport through mitochondrial complex I, resulting in the generation of large amounts of ROS. Our experiments confirmed that silibinin enhances the function of mitochondrial complex II while inhibiting the function of complex I, which can reduce the production of ROS generated by reverse electron flow, thus protecting the mitochondria. In addition, NAD + acts as a cofactor in various metabolic pathways, such as glycolysis, fatty acid oxidation, and the tricarboxylic acid cycle, participating in cellular energy metabolism (Zhang et al., 2024; Aggarwal et al., 2022). NADH is the reduced form of NAD+ and serves as a substrate for mitochondrial respiratory chain NADH dehydrogenase (Dall et al., 2022). Both NAD+ and NADH play important coenzyme roles in redox reactions, and a decrease or imbalance in their ratio may disrupt the flow of reactions in these pathways, leading to imbalanced cellular metabolism (Guarino and Dufour, 2019). It has been found that various natural products can prevent and treat various diseases by regulating NAD + metabolism (Guo et al., 2023). Our study showed that PM2.5 treatment significantly reduced the levels of total NAD, NAD+, and NADH, as well as total NADP+ and NADPH, while silibinin treatment restored their levels. These results indicate that mitochondria play a crucial role in the accumulation of triglycerides in the liver caused by PM2.5 exposure and the therapeutic effect of silibinin.

In conclusion, we can draw a conclusion that silibinin can inhibit the accumulation of triglycerides in the liver caused by PM2.5 b y protecting mitochondrial function and reducing ROS production. These findings expand the pharmacological understanding of silibinin and provide some ideas for the prevention and treatment of liver damage caused by atmospheric particulate matter. However, the limitation of this study is that we focused on the redox state of cells and organisms in this paper, and therefore measured the levels of ROS, NAD+/NADH, and NADP+/NADPH in cells. However, the spatial distribution of redox substances in cells is not uniform (Zhao et al., 2015). Some researchers have mentioned the concept of compartmentalization of redox zones (Zou et al., 2018), pointing out that the redox state between organelles is different. Therefore, more research is needed to use more accurate methods to detect the redox state of subcellular structures after PM2.5 exposure.

## Conclusion

This study’s findings suggest that PM2.5-induced liver fat accumulation involves a series of physiological changes, including alterations in ROS, inflammation and anti-inflammatory factors, MDA/GSH/SOD, NAD+/NADH and NADP+/NADPH, and mitochondrial complex functionality. Importantly, silibinin inhibits liver fat accumulation caused by PM2.5 by protecting the functionality of mitochondrial complexes. The key target proteins that directly interact with silibinin and contribute to the observed lipid-lowering effects is succinate dehydrogenase. In addition, it is still necessary to conduct more research. Therefore, the discovery of drugs capable of improving mitochondrial function is promising for providing effective treatment strategies for PM 2.5-induced pathological liver injury.

## Figures and Tables

**Table:** 

**PM2.5**	Fine Particulate Matter
**ROS**	Reactive Oxygen Species
**TG**	Triglycerides
**TC**	Total Cholesterol
**AST**	Aspartate Aminotransferase
**ALT**	Alanine Aminotransferase
**MDA**	Malondialdehyde
**GSH**	Glutathione
**SOD**	Superoxide Dismutase
**SDHA**	Succinate Dehydrogenase Complex Subunit A
**SDHB**	Succinate Dehydrogenase Complex Subunit B
**SDHC**	Succinate Dehydrogenase Complex Subunit C
**SDHD**	Succinate Dehydrogenase Complex Subunit D
**ASP**	Aspartic Acid
**GLN**	Glutamine
**THR**	Threonine
**PRO**	Proline
**GLU**	Glutamic Acid
**ASN**	Asparagine
**TRP**	Tryptophan
**NDUFA2**	Mitochondrial Complex I Subunit 2
**NDUFA7**	Mitochondrial Complex I Subunit 7
**NDUFA8**	Mitochondrial Complex I Subunit 8
**NDUFS6**	Mitochondrial Complex I Subunit 6
**CYCS**	Cytochrome c
**COX6B1**	Cytochrome c Oxidase Subunit 6B1
**ATP4A**	ATP Synthase F0 Subunit 4
**ATP5ME**	ATP Synthase F1 Subunit epsilon
**UQCRQ**	Ubiquinol-Cytochrome c Reductase Binding Protein
**UQCRC1**	Ubiquinol-Cytochrome c Reductase Core Protein 1
**UQCRFS1**	Ubiquinol-Cytochrome c Reductase Rieske Iron-Sulfur Polypeptide 1
**ATP6V0C**	ATPase H + Transporting V0 Subunit C
**ATP6V1G2**	ATPase H + Transporting V1 Subunit G2
**Nrf2**	Nuclear factor erythroid 2-related factor 2
**HO-1**	Heme oxygenase-1
**NQO-1**	NAD(P)H Quinone Oxidoreductase 1
**GCLC**	Glutamate-cysteine ligase catalytic subunit
**GST**	Glutathione S-transferase
**FADH2**	Flavin Adenine Dinucleotide
**ATP**	Adenosine Triphosphate
**NASH**	Non-Alcoholic Steatohepatitis
**NAFLD**	Non-Alcoholic Fatty Liver Disease
**NLRP3**	NOD-Like Receptor Family, Pyrin Domain Containing 3
**TNF-α**	Tumor Necrosis Factor-alpha
**IL-1α**	Interleukin-1α
**IL-1β**	Interleukin-1β
**IL-8**	Interleukin-8
**NADP+**	Nicotinamide Adenine Dinucleotide Phosphate
**NADPH**	Nicotinamide Adenine Dinucleotide Phosphate, reduced form
**NAD+**	Nicotinamide Adenine Dinucleotide
**NADH**	Nicotinamide Adenine Dinucleotide, reduced form

## Data Availability

The original contributions presented in the study are included in the article/Supplementary Material, further inquiries can be directed to the corresponding authors.

## Supplementary material

SUPPLEMENTARY FIGURE S1 PM2.5 induces disruption of serum biomarkers levels. **(A)** Liver TG levels after treatment with high (92.4 mg/kg), medium (46.2 mg/kg), and low (23.1 mg/kg) concentrations of silibinin capsules. **(B)** Liver TC levels after treatment with high (92.4 mg/kg), medium (46.2 mg/kg), and low (23.1 mg/kg) concentrations of silibinin capsules. n = 6 replicates. **(C)** Serum ALT levels. **(D)** Serum AST levels. **(E)** Serum HDL-C levels. **(F)** Serum LDL-C levels. **(G)** Serum TC levels. **(H)** Serum TG levels. n = 6 replicates. The data mentioned earlier were presented as means ± SEMs and subjected to one-way ANOVA for analysis. *p < 0.05 vs.NC. ^#^p < 0.05 vs. PM2.5.

SUPPLEMENTARY FIGURE S2 Real-time PCR validation of the regulation of pro-inflammatory genes and antioxidant genes across four groups in mouse livers. **(A)** qPCR analyses of pro-inflammatory factors *IL-1α, IL-1β, NLRP3, TNF-α*, and *IL-8* levels in mouse livers among the four groups. n = 4 replicates. **(B)** qPCR analyses of antioxidants *Nrf2, HO-1, NQO-1*, *GCLC*, and *GST* levels in mouse livers among the four groups. n = 4 replicates. The data mentioned earlier were presented as means ± SEMs and subjected to Student’s t-test for analysis. *p < 0.05 vs. Control; ^#^p < 0.05 vs. PM2.5.

SUPPLEMENTARY FIGURE S3 The PM2.5 particles in an aggregated state exhibit a uniform particle distribution after 72 h of ultrasonic treatment. **(A,B)** Images show the difference between PM2.5 before **(A)** and after **(B)** sonicationand statistical results **(C)**. n = 9 replicates. The data mentioned earlier were presented as means ± SEMs and subjected to Student’s t for analysis *p < 0.05 vs. the unsonicated.

SUPPLEMENTARY FIGURE S4 Silibinin treatment affects the levels of NAD+/NADH and NADP+/NADPH in Hep G2 cells induced by PM2.5. **(A–D)** Histograms display the levels of total NAD **(A)**, NAD+ **(B)**, NADH **(C)** and NADH/NAD+ **(D)** in each group. n = 3 replicates. **(E–H)** Histograms display the levels of total NADP **(E)**, NADP+ **(F)**, NADPH **(G)** and NADP+ / NADPH **(H)** in each group. n = 6 replicates. The data mentioned earlier were presented as means ± SEMs and subjected to one-way ANOVA for analysis *p < 0.05 vs.Control; ^#^p < 0.05 vs. PM2.5.

SUPPLEMENTARY FIGURE S5 The effects of Silibinin on mitochondrial Complex I and II activity. **(A,B)** Complex I respiratory function in Hep G2 liver cells (permeabilized with Digoxin) under treatment with Silibinin illustrated by representative results (left) and supported by statistical findings (right). n = 5 replicates. **(C,D)** Complex II respiratory function in Hep G2 liver cells (permeabilized with Digoxin) under the influence of silibinin treatment is elucidated through representative (left) and statistical (right) results. n = 5 replicates. The data mentioned earlier were presented as means ± SEMs and subjected to Student’s t for analysis *p < 0.05 vs. Control.
