# Supplementary material for: Silibinin inhibits PM2.5-induced liver triglyceride accumulation through enhancing the function of mitochondrial Complexes I and II
Source: Front Pharmacol. 2024 Sep 16;15:1435230. doi: 10.3389/fphar.2024.1435230 (PMC11440093; doi:10.3389/fphar.2024.1435230)
Supplement: Supplementary file 1 [file DataSheet1.zip › Supplemental Materials/Graphical abstract.PDF]

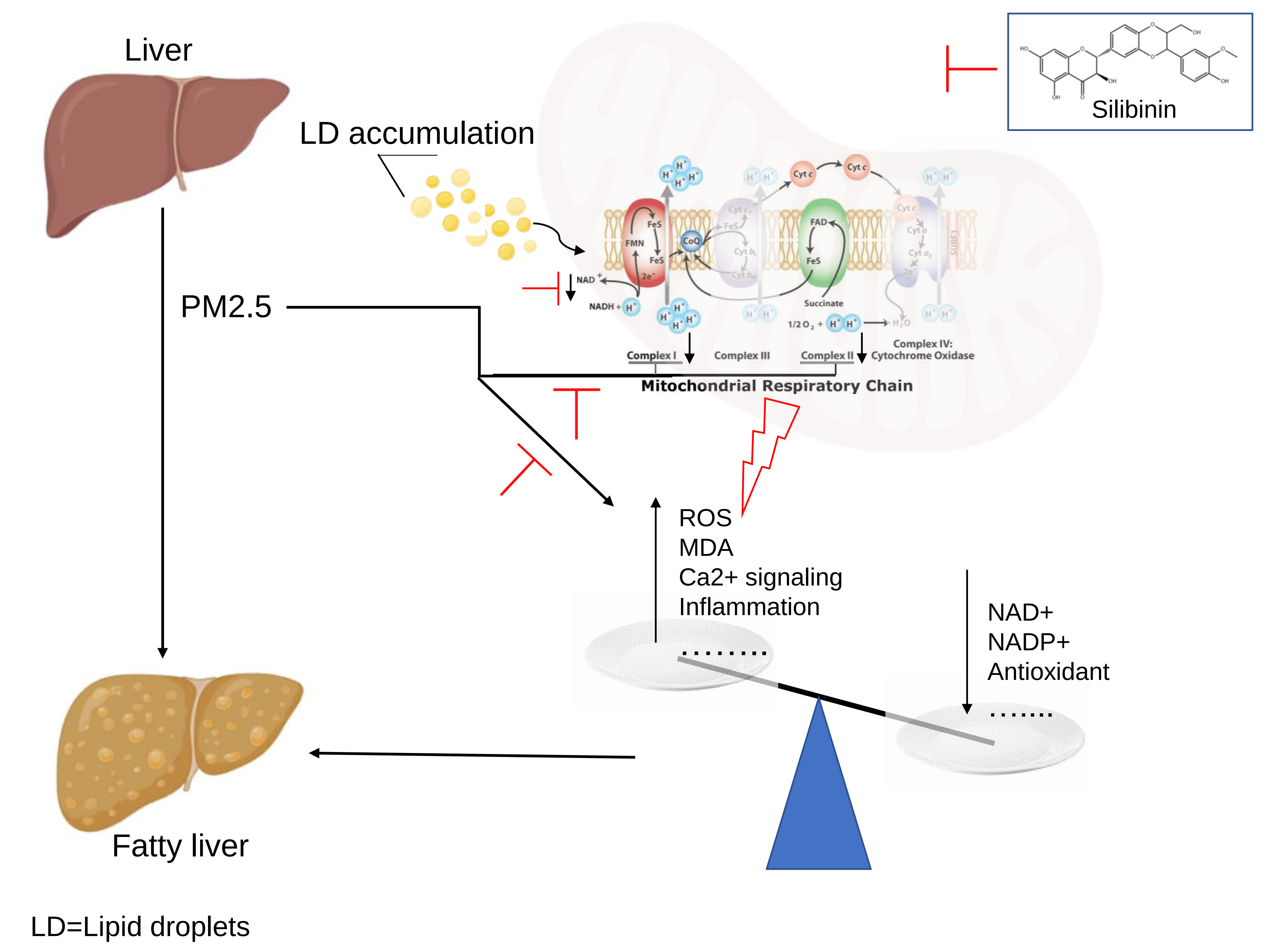
Liver
LD accumulation
Silibinin
PM2.5
NAD+
NADH + H+
FMN
FeS
CoQ
Cyt c
FAD
Succinate
1/2 O2 + H+ H+ → H2O
Complex I
Complex III
Complex II
Complex IV:
Cytochrome Oxidase
Mitochondrial Respiratory Chain
ROS
MDA
Ca2+ signaling
Inflammation
NAD+
NADP+
Antioxidant
Fatty liver
LD=Lipid droplets
