# Supplementary material for: Silibinin inhibits PM2.5-induced liver triglyceride accumulation through enhancing the function of mitochondrial Complexes I and II
Source: Front Pharmacol. 2024 Sep 16;15:1435230. doi: 10.3389/fphar.2024.1435230 (PMC11440093; doi:10.3389/fphar.2024.1435230)
Supplement: Supplementary file 1 [file DataSheet1.zip › Supplemental Materials/Table (Primer Sequences from the Article).docx]

Table 1 Primer Sequences

| Gene Names | Sequence |
| --- | --- |
| Human UQCRQ-F | CGCGAGTTTGGGAATCTGAC |
| Human UQCRQ-R | TAGTGAAGACGTGCGGATAGG |
| Human ATP6V0C-F | ATGTCCGAGTCCAAGAGCG |
| Human ATP6V0C-R | GGCCGTAGATGGCGATGAT |
| Human NDUFA8-F | CCCAACAAGGAGTTTATGCTCT |
| Human NDUFA8-R | CACAGTGACGTTTTATCTGCCT |
| Human ATP6V1G2-F | CAGTCAGTCCCAAGGTATCCA |
| Human ATP6V1G2-R | CTGCGGTATTGCTCCACCTC |
| Human UQCRFS1-F | CTGAATACCGCCGCCTTGAA |
| Human UQCRFS1-R | ATGCGACACCCACAGTAGTTA |
| Human UQCRC1-F | GGGGCACAAGTGCTATTGC |
| Human UQCRC1-R | GTTGTCCAGCAGGCTAACC |
| Haman IP3R-F | GCGGAGGGATCGACAAATGG |
| Haman IP3R-R | TGGGACATAGCTTAAAGAGGCA |
| Haman SERCA2-F | ATGGGGCTCCAACGAGTTAC |
| Haman SERCA2-R | TTTCCTGCCATACACCCACAA |
| Haman Calpain-2-F | GAAGCGTCCCACGGAACTG |
| Haman Calpain-2-R | GTGCAGGAGGGTGTCGTTG |
| Haman PERK-F | ACGATGAGACAGAGTTGCGAC |
| Haman PERK-R | ATCCAAGGCAGCAATTCTCCC |
| Haman CHOP-F | GGAAACAGAGTGGTCATTCCC |
| Haman CHOP-R | CTGCTTGAGCCGTTCATTCTC |
| Haman CYP1A1-F | TCGGCCACGGAGTTTCTTC |
| Haman CYP1A1-R | GGTCAGCATGTGCCCAATCA |
| Haman CYP1A2-F | CTGGGCACTTCGACCCTTAC |
| Haman CYP1A2-R | TCTCATCGCTACTCTCAGGGA |
| Haman CYP1B1-F | TGAGTGCCGTGTGTTTCGG |
| Haman CYP1B1-R | GTTGCTGAAGTTGCGGTTGAG |
| Haman TiPARP-F | AGAACGAGTGGTTCCAATCCA |
| Haman TiPARP-R | TGGGTGCAAAAGATCAGTCTG |
| Haman CD38-F | CAACTCTGTCTTGGCGTCAGT |
| Haman CD38-R | CCCATACACTTTGGCAGTCTACA |
| Haman AHRR-F | CAAATCCTTCCAAGCGGCATA |
| Haman AHRR-R | CGCTGAGCCTAAGAACTGAAAG |
| Human IL-1α-F | AGATGCCTGAGATACCCAAAACC |
| Human IL-1α-R | CCAAGCACACCCAGTAGTCT |
| Human IL-1β-F | ATGATGGCTTATTACAGTGGCAA |
| Human IL-1β-R | GTCGGAGATTCGTAGCTGGA |
| Human NLRP3-F | CGTGAGTCCCATTAAGATGGAGT |
| Human NLRP3-R | CCCGACAGTGGATATAGAACAGA |
| Human TNF-α-F | ACCTCCGAGATGACACCATCA |
| Human TNF-α-R | GGCACTCTGGCACATATTCAC |
| Human IL-8-F | ACTGAGAGTGATTGAGAGTGGAC |
| Human IL-8-R | AACCCTCTGCACCCAGTTTTC |
| Human Nrf2-F | TCAGCGACGGAAAGAGTATGA |
| Human Nrf2-R | CCACTGGTTTCTGACTGGATGT |
| Human HO-1-F | AAGACTGCGTTCCTGCTCAAC |
| Human HO-1-R | AAAGCCCTACAGCAACTGTCG |
| Human NQO-1-F | GAAGAGCACTGATCGTACTGGC |
| Human NQO-1-R | GGATACTGAAAGTTCGCAGGG |
| Human GCLC-F | CATTTACAGCCTTACTGGGAGG |
| Human GCLC-R | ATGCAGTCAAATCTGGTGGCA |
| Human GST-F | TCTGGAAAAGATCGCAACGC |
| Human GST-R | GCCCAAAGGCTCCGTATCTG |
| Human beta-actin-F | CATGTACGTTGCTATCCAGGC |
| Human beta-actin-R | CTCCTTAATGTCACGCACGAT |
| Mouse IL-1α-F | CGAAGACTACAGTTCTGCCATT |
| Mouse IL-1α-R | GACGTTTCAGAGGTTCTCAGAG |
| Mouse IL-1β-F | GCAACTGTTCCTGAACTCAACT |
| Mouse IL-1β-R | ATCTTTTGGGGTCCGTCAACT |
| Mouse NLRP3-F | ATTACCCGCCCGAGAAAGG |
| Mouse NLRP3-R | TCGCAGCAAAGATCCACACAG |
| Mouse TNF-α-F | CCCTCACACTCAGATCATCTTCT |
| Mouse TNF-α-R | GCTACGACGTGGGCTACAG |
| Mouse IL-8-F | CAAGGCTGGTCCATGCTCC |
| Mouse IL-8-R | TGCTATCACTTCCTTTCTGTTGC |
| Mouse Nrf2-F | TCTTGGAGTAAGTCGAGAAGTGT |
| Mouse Nrf2-R | GTTGAAACTGAGCGAAAAAGGC |
| Mouse HO-1-F | AAGCCGAGAATGCTGAGTTCA |
| Mouse HO-1-R | GCCGTGTAGATATGGTACAAGGA |
| Mouse NQO-1-F | AGGATGGGAGGTACTCGAATC |
| Mouse NQO-1-R | AGGCGTCCTTCCTTATATGCTA |
| Mouse GCLC-F | GGGGTGACGAGGTGGAGTA |
| Mouse GCLC-R | GTTGGGGTTTGTCCTCTCCC |
| Mouse GST-F | CTCAGGCAGCTCATGGACAAT |
| Mouse GST-R | GTTATCCTCTGGAATGCGGTC |
| Mouse NDUFA2-F | TTGCGTGAGATTCGCGTTCA |
| Mouse NDUFA2-R | TTGCGTGAGATTCGCGTTCA |
| Mouse COX6B1-F | ACTACCTGGACTTCCACCG |
| Mouse COX6B1-R | ACCCATGACACGGGACAGA |
| Mouse NDUFA7-F | TCCGCTACTCGCGTTATCCA |
| Mouse NDUFA7-R | GATTGAGGGAGGCACAACTTC |
| Mouse NDUFA8-F | GGAGCTGCCAACTCTGGAAG |
| Mouse NDUFA8-R | CCAGCGGCACAGCATAAAC |
| Mouse NDUFS6-F | ATACCGGCCAGGTAACGATG |
| Mouse NDUFS6-R | AGGCTGTTGTGCTATCAAATCAA |
| Mouse CYCS-F | CCAAATCTCCACGGTCTGTTC |
| Mouse CYCS-R | ATCAGGGTATCCTCTCCCCAG |
| Mouse ATP4A-F | GATGGAGATTAACGACCACCAG |
| Mouse ATP4A-R | ACGGGCAAACTTCACATACTC |
| Mouse ATP5ME-F | GTTCAGGTCTCTCCACTCATCA |
| Mouse ATP5ME-R | CGGGGTTTTAGGTAACTGTAGC |
| Mouse beta-actin-F | GGCTGTATTCCCCTCCATCG |
| Mouse beta-actin-R | CCAGTTGGTAACAATGCCATGT |
